# Discovery and characterization of an anti-*Neisseria gonorrhoeae* NGO_1985 monoclonal antibody and cognate antigen

**DOI:** 10.3389/fmicb.2026.1830193

**Published:** 2026-06-24

**Authors:** Pardis Mokhtary, Samuele Stazzoni, Eleonora Marini, Marco Troisi, Tomi T. Airenne, Mikko Huhtala, Giuseppe Maccari, Lucia Eleonora Fontana, Nathalie Norais, Laura Tinti, Vittoria Cicaloni, Laura Salvini, Monica Fabbrini, Tiina A. Salminen, Emanuele Andreano, Claudia Sala, Rino Rappuoli

**Affiliations:** 1 Monoclonal Antibody Discovery Laboratory, Fondazione Toscana Life Sciences, Siena, Italy; 2 Department of Biotechnology, Chemistry and Pharmacy, University of Siena, Siena, Italy; 3 Structural Bioinformatics Laboratory, Biochemistry, Faculty of Science and Engineering, Åbo Akademi University, Turku, Finland; 4 InFLAMES Research Flagship Center, Åbo Akademi University, Turku, Finland; 5 Data Science for Health Laboratory, Fondazione Toscana Life Sciences, Siena, Italy; 6 GSK, Siena, Italy; 7 Mass Spectrometry Unit, Fondazione Toscana Life Sciences, Siena, Italy; 8 Fondazione Biotecnopolo di Siena, Siena, Italy

**Keywords:** Bexsero, monoclonal antibodies, *Neisseria gonorrhoeae*, NGO_1985, Reverse Vaccinology 2.0, YraP

## Abstract

Gonorrhea, caused by the Gram-negative bacterium *Neisseria gonorrhoeae* (*Ng*), affects millions of people globally. The absence of a vaccine and the rising antibiotic resistance complicate infection control, highlighting the urgent need for new prophylactic and therapeutic strategies. Since partial protection against gonorrhea has reportedly been linked to vaccination with the anti-meningococcal 4CMenB vaccine, we utilized Reverse Vaccinology 2.0 to identify gonococcal antigens through characterization of monoclonal antibodies (mAbs) produced by memory B cells (MBCs) following vaccination of volunteers with 4CMenB. A mAb, named mAb_4, with cross-reactivity among *Ng* strains was selected for deeper investigation. Genetic and biochemical approaches revealed that the cognate antigen was NGO_1985, a protein involved in outer membrane biogenesis and stability. Notably, NGO_1985 is the ortholog of the *N. meningitidis* (*Nm*) GNA_2091 protein, which is included as a fusion protein in the 4CMenB vaccine and contributes to stability and immunogenicity. The NGO_1985 epitope recognized by mAb_4 was predicted *in silico* and experimentally confirmed by Hydrogen Deuterium Exchange Mass Spectrometry. This study enhances our understanding of the molecular basis for the cross-reaction/protection conferred by 4CMenB and might guide future developments of antibody cocktail therapeutics, protein-based vaccines, and *in vivo* research.

## Introduction

Antibacterial resistance is a significant global public health concern contributing to an estimated 4.95 million deaths in 2019, with a disproportionate number occurring in low- and middle-income countries (LMIC) (Antimicrobial Resistance Collaborators, 2022).

*Neisseria gonorrhoeae* (*Ng*), also known as Gonococcus, is the Gram-negative diplococcus responsible for the sexually transmitted disease gonorrhea which has long posed a public health challenge (Zhong et al., 2024) with 82 million new cases reported in 2020 (World Health Organization, 2024). In recent years, the emergence of multi-drug resistant *Ng* strains have led to treatment failures worldwide (Nguyen et al., 2014) and prompted the World Health Organization (WHO) to classify *Ng* as a high-priority pathogen in the 2024 Bacterial Priority Pathogen List (BPPL) (Sati et al., 2025). While *Ng* infections are typically not fatal, they can cause severe long-term health issues including infertility, pelvic inflammatory disease, chronic pelvic pain, and ectopic pregnancies in women, as well as epididymitis in men (Workowski et al., 2021). Furthermore, a higher prevalence of *Ng* is associated with an elevated risk of contracting and spreading human immunodeficiency virus 1 (HIV-1) and other sexually transmitted diseases (STDs) (Workowski et al., 2021; Cohen, 1998; Fleming and Wasserheit, 1999). To date, there are no approved vaccines for *Ng* infections. Efforts to develop an effective prophylactic strategy have been hindered by *Ng* ability to alter its surface antigens and potential vaccine targets (Maurakis and Cornelissen, 2022).

Recent findings have renewed optimism in gonococcal vaccine research. Retrospective studies have shown partial protection against *Ng* infection by the *Neisseria meningitidis* (*Nm*) serogroup B (MenB) Outer Membrane Vesicle (OMV)-based vaccine (Semchenko et al., 2019; Paynter et al., 2019). However, a recent clinical trial showed that the 4CMenB vaccine did not reduce gonorrhea incidence in high-risk individuals (NSW, 2026; CIDRAP, 2026; Thng et al., 2026). 4CMenB is a multi-component vaccine that protects against *Nm* and may also protect against gonorrhea (Paynter et al., 2019). Observational studies conducted in the US and Australia have corroborated prior data from Canada, Cuba, and Norway, showing 30–46% protection against *Ng* infection by the OMV-based MenB vaccine (MeNZB) (Bruxvoort et al., 2023; Abara et al., 2022). This information suggests that a *Ng* vaccine is attainable and that MenB OMV-based vaccines may contain antigens capable of eliciting cross-reactive immunity between the two species, *Nm* and *Ng*, which share 80–90% identity at the genome level (Tinsley and Nassif, 1996). Shared antigens between *Nm* and *Ng* may be targets of cross-reactive antibodies induced by 4CMenB thereby justifying the search for cross-protective immunogens for understanding broad protection and guiding vaccine development. 4CMenB includes the MeNZB OMV component and three recombinant antigens: NadA (Neisserial adhesin A), fHbp (factor H binding protein) fused to GNA2091, and NHBA (Neisserial heparin binding antigen) fused to GNA1030 (Rappuoli et al., 2018; Marjuki et al., 2019). It has been suggested that other subdominant MenB antigens might be responsible for the observed cross-protection as *Ng* does not express the PorA immunodominant protective antigen present in the meningococcal OMV vaccination (Semchenko et al., 2019; Marjuki et al., 2019). Our recent research demonstrated that immunity elicited by the 4CMenB vaccine against *Ng* is largely due to the PorB protein and the lipooligosaccharide (LOS), two antigens shared by *Ng* and *Nm* (Troisi et al., 2025). Inspired by this discovery, we sought for additional targets capable of eliciting an immune response against *Ng* that could be harnessed in protein-based vaccines.

Proteins located in the bacterial cell envelope and membrane vesicles are promising targets for prevention and therapy of gonorrhea, and particularly those involved in outer membrane structure and assembly may be accessible to antibodies (Zielke et al., 2014). Among these proteins, the Bam complex is crucial for assembling *β*-barrel outer membrane proteins (OMPs), which can act as channels for the transport of nutrients and other proteins across the membrane, into the outer membrane (OM) (Malinverni and Silhavy, 2011; Lundquist et al., 2021). Prior research has established that the Bam complex consists of BamA, a *β*-barrel transmembrane protein, and four lipoproteins: BamB, BamC, BamD, and BamE. Given its essential role in outer membrane biogenesis, the Bam complex is critical for bacterial viability. Disruption of this system has the potential to impair membrane integrity, suggesting that components of the Bam complex may represent biologically relevant targets for antibody-mediated interference (Malinverni and Silhavy, 2011; Stenberg et al., 2005; Wu et al., 2005; Sklar et al., 2007).

NGO_1985 has previously been referred to as BamG in two gonococcal studies (Baarda et al., 2018; El-Rami et al., 2019). However, its mechanistic integration into the Bam complex has not been demonstrated. More recently, BamG in Bacteroidetes (Silale et al., 2025) was shown to represent a structurally characterized, integral *β*-barrel component of the Bam complex, distinct from the *Neisseria* YraP homolog NGO_1985. NGO_1985 has been discovered in a proteome-derived vaccine candidate investigation and was later recognized as an accessory lipoprotein within the Bam complex (Baarda et al., 2018; El-Rami et al., 2019).

Here, we report on the isolation and characterization of a human monoclonal antibody (mAb), named mAb_4, isolated from people immunized with the 4CMenB vaccine. The antibody recognizes gonococcal and meningococcal NGO_1985, a protein which was included in 4CMenB as a fusion protein because of its ability to stabilize the vaccine and promote immunogenicity in animal models. This provides a direct link between vaccination and the potential generation of antibodies recognizing this conserved antigen. These findings demonstrate that 4CMenB vaccination can elicit antibodies targeting conserved, non-classical antigens shared between the two species. This study advances our understanding of the molecular bases of the cross-reactivity provided by 4CMenB and may guide the development of future antibody cocktail therapeutics, protein-based vaccines, and *in vivo* research.

## Materials and methods

### Donor recruitment, PBMC collection, and mAb isolation

In a previous study (Troisi et al., 2025) we had discovered anti-*Ng* mAbs from healthy volunteers who had received two doses of the 4CMenB vaccine. The study had received approval from the local Ethics Committee (AOU Senese, Parere nr. 13946_2018) and was conducted in accordance with good clinical practice and the declaration of Helsinki (European Council 2001, US Code of Federal Regulations, ICH 1997). Peripheral Blood Mononuclear Cells (PBMCs) had been collected 28 days after the booster dose and Memory B Cells (MBCs) isolated by single-cell sorting upon staining as follows: CD19 BV421 (BD), IgM PerCP-Cy5.5 (BD), CD27 PE (BD), IgD Alexa Fluor 700 (BD), CD3 PE-Cy7 (BioLegend), CD14 PE-Cy7 (BioLegend), CD56 PE-Cy7 (BioLegend) and Alexa Fluor 488-labeled MenB OMVs (Troisi et al., 2025). The sorting strategy, which resulted in the isolation of 3,080 IgG^+^ and IgA^+^ MBCs that exhibited binding to 4CMenB outer membrane vesicles (OMVs), is summarized in Supplementary Figure S1. ELISA experiments (twice the value of the background was the threshold for positivity) had pinpointed mAbs that reacted against *Ng* strains (Troisi et al., 2025). Among the positive hits, mAb_4 was selected for further studies as described in this work.

### Bacterial strains and culture conditions

*Ng* strains used in this work are listed in Supplementary Table S1. Bacteria were cultivated in gonococcal (Gc) liquid medium plus 1% (v/v) Isovitalex (BD Biosciences, Franklin Lakes, NJ, USA), or on gonococcal agar (GCA) supplemented with 1% (v/v) Isovitalex at 37 °C under 5% CO_2_.

### Quantitative enzyme-linked immunosorbent assay (ELISA)

To assess the amount of mAb in cell supernatants, ELISA was carried out in 384-well plates. Plates were pre-coated with 2 μg/mL goat anti-human IgG (SouthernBiotech) at 4 °C overnight. Bovine Serum Albumin (BSA) 1% (w/v) - phosphate-buffered saline (PBS) 1X-Tween 0.05% (v/v) was used as a blocking agent for 1 h at 37 °C, followed by the addition of mAb supernatants diluted 1:10. Positive control was 10 μg/mL human IgG-UNLB (SouthernBiotech). After 1 h at 37 °C, mAbs were washed and a secondary goat anti-human IgG-Alkaline phosphatase (SouthernBiotech) antibody was added diluted 1:2,000 in PBS1X-BSA 1% + 0.05% Tween. The alkaline phosphatase substrate p-Nitrophenyl Phosphate (PNPP) (Sigma-Aldrich) was added and incubated for 30 min at RT before measuring the luminescence signal with a Varioskan LUX multimode microplate reader (Thermo Fisher Scientific, Waltham, MA, USA). All samples were tested in duplicate. Background was subtracted, and data were expressed as relative signal intensity. Samples were considered positive when signals exceeded 2-fold above background.

### Enzyme-linked immunosorbent assay on whole-bacterial cells

Following growth to the exponential phase, bacteria were resuspended in the same volume of PBS after being centrifuged for 5 minutes at 4,500 xg. After incubation at 37 °C, 5% CO_2_ for 30 min, a washer dispenser (BioTek EL406, Agilent Technologies, US) automatically washed plates with bacteria twice with PBS, Tween-20 0.05%, 70 μL per well. Bacteria were fixed at RT for 30 min with 0.5% formaldehyde. To avoid unspecific binding, wells were saturated with PBS, and BSA 1 in 75 μL. After incubation for 1 h at 37 °C, wells were washed, then primary antibodies were added in a 1:5 ratio in PBS, BSA 1%, Tween20 0.05% in 30 μL/well final volume and incubated for 1 h at 37 °C without CO_2_. Following the washing step, 30 μL of alkaline phosphatase-conjugated goat anti-human IgG (Sigma-Aldrich, US) or IgG (Southern Biotech) secondary antibodies were added. After a final wash, pNPP (p-nitrophenyl phosphate) (Sigma-Aldrich) was employed to detect mAb binding. The Varioskan Lux Reader (Thermo Fisher Scientific, US) recorded 405 nm absorbance. Samples were considered positive if they had an optical density (OD) at 405 nm (OD_405_) three times the blank.

### Binding characterization by cytofluorimetry

50 μL of the bacterial suspension (Log phase bacteria) diluted in PBS and BSA 1% (w/v) to an OD_600_ of 0.2 were added to Corning 96-well round bottom tissue culture-treated microplates. After centrifuging the plate at 4,500 xg for 5 min, bacteria were resuspended in 50 μL of PBS containing BSA 1% and 10 μg/mL of primary antibodies. The centrifugation procedure was repeated after one hour of incubation at 37 °C with 5% CO_2_. Bacteria were suspended in 50 μL PBS, 1% BSA, and diluted 1/2000 Alexa Fluor 488 goat secondary immunoglobulin G (IgG) antibody (Thermo Fisher Scientific). Following centrifugation, bacteria were fixed for 30 min at room temperature in 0.5% formaldehyde. After fixation, the bacterial cultures were centrifuged for 5 min at 4,500 xg. The pellet was then re-suspended in 50 μL PBS. BD FACS Canto II flow cytometer was used to analyze samples. 10,000 counts were considered for each sample. The analysis was conducted using FlowJo version 10.

### Visual binding assay with confocal microscopy

*Ng* strains were cultured at 37 °C in 5% CO₂ under shaking conditions and collected at mid-logarithmic phase (OD₆₀₀ ≈ 0.5), which was usually reached after ~15 h. PBS-BSA 1% buffer was used to prepare a mirror plate containing mAbs at 10 μg/mL. Bacteria were centrifuged at 4500 × g for 5 min, resuspended in PBS and diluted to OD_600_ 0.1. Next, 50 μL of the bacterial suspension were transferred to a PhenoPlate 96-well microplate in triplicate wells and incubated at 37 °C with 5% CO_2_ for 30 min. Following the removal of the supernatant, 150 μL of PBS was used to wash the samples. To fix the bacteria, 0.5% paraformaldehyde was used. Following a half-hour incubation at 37 °C with 5% CO_2_, wells were washed twice with 150 μL PBS and then 50 μL from the mirror plate were added and incubated for one hour at 37 °C. After washing, samples were incubated for 30 min at 37 °C with Alexa 488 anti-human (1:2000) in PBS-BSA 1% buffer. The plate was once again washed with 150 μL PBS and stained with DAPI. Samples were incubated for 30 min at 4 °C and imaged using the Opera Phenix High-Content Screening System (Revvity) microscope at 63x magnification and 1.1 numerical aperture. For each well 16 fields of view were acquired, and Z-stacks were composed of 13 planes.

### nOMVs (native outer membrane vesicles) production from *N. gonorrhoeae*

*Ng* strains were streaked, inoculated and grown to OD_600_ 0.2 in a one-liter baffled flask at 37 °C at 150 rpm for about 3 h (stationary phase) and then 5 g/L of Na-lactate was added for 3 h. The culture supernatant was collected by centrifugation at 8000 xg at 4 °C for 30 min, then passed through Nitrocellulose MF-Millipore™ membrane filters (0.22 μm) to remove any remaining cells and cellular debris. The filtered supernatant was then ultracentrifuged to pellet OMVs by using a 45-Ti fixed angle rotor and Thermo Scientific SORVALL WX Ultra at 170,000 xg for 3 h, at 4 °C. Pelleted OMVs were washed twice in PBS and resuspended in 1 mL of PBS. nOMV proteins were quantified by Lowry assay and the protein pattern was assessed by SDS-PAGE.

### SDS-PAGE and immunoblotting

100 μg of OMV proteins were loaded on NuPAGE 4–12% Bis-Tris Gels (Thermo Fisher Scientific) and gels were run in MES SDS Running Buffer 1X. Samples were transferred by using iBlot 2 Transfer Stacks in the Invitrogen™ iBlot™ 2 Gel Transfer Device. Blocking of the PVDF membrane was performed in blocking buffer (20 mM TBS, 0.05% Tween 20, Non-fat dry milk). Antibodies at the concentration of 10 μg/mL in blocking buffer were added and incubated for 24 h at 4 °C. Membranes were washed three times, each time 5 minutes in TBS (20 mM TBS, 0.05% Tween 20). A secondary antibody (anti-human IgG) diluted 1/75,000 in blocking buffer was added and incubated for one hour at 4 °C. Membranes were washed again in the same manner as before. The membrane was then incubated with the SuperSignal™ Western Blot Enhancer kit reagent and developed using the Invitrogen™ iBright™ FL1500 Imaging System instrument.

### Lysed OMVs and immunoprecipitation/mass spectrometry (IP/MS)

NP-40 lysis buffer (20 mM Tris–HCl pH 8, 165 mM NaCl, 1% Nonidet P-40 (NP-40), and 5 mM EDTA) is nonionic and mild. After 10 min of NP-40 treatment on ice, the OMVs were centrifuged and the supernatant was collected for immunoprecipitation experiments. Dynabeads Protein G (50 mL per sample) was transferred to a tube at RT and placed on a magnet. After 1 min, the supernatant was discarded and beads were resuspended in 0.5 mL of PBS pH 7.4 w/Tween 20 (0.05%). Candidate mAbs (1–10 mg/mL) were added and incubated at RT for 1 h with gentle shaking. 10–50 μg/mL of lysed OMVs were added to the beads coupled with the mAb at 4 °C overnight with gentle shaking. On the following day, samples were washed and eluted in Tris-Glycine pH 2.7. Neutralization was accomplished in 1 M Tris–HCl pH 9.0. Immunoprecipitated samples were first reduced using dithiothreitol (DTT) and alkylated with iodoacetamide at room temperature before protein precipitation obtained adding. 80% methanol/20% chloroform. Precipitates were pelleted for 2 min at 4 °C. The pellets were dried and resuspended in 6 M urea. Peptide digestion was carried out using trypsin (Promega) overnight at 37 °C. Tryptic peptides were desalted and dried in a vacuum centrifuge prior to mass spectrometry analysis. The samples were then resuspended in water and 0.1% trifluoracetic acid (TFA) and analysed by mass spectrometry. LC–MS/MS analyses were performed using Q-Exactive HF-X Orbitrap mass spectrometer (Thermo Fisher Scientific). The peptide separation was carried out using a PepMap RSLC C18 column, 75 um × 15 cm, 2 μm, 100 Å (Thermo Fisher) at a flow rate of 300 nL/min. The mobile phases A and B used for the analysis were 0.1% formic acid in water and 0.1% formic acid in acetonitrile, respectively. The gradient started with 5% of B and then it was increased to 90% in 120 min. The experiment was performed using a data dependent analysis (DDA) setting to select the “top twenty” most-abundant ions for MS/MS analysis. Protein identification was performed by Proteome Discoverer 2.5 (Thermo Scientific).

### NGO_1985 expression

The high copy number vector pET28a was selected for the expression of NGO_1985. The DNA sequence encoding the protein was synthesized by Twist and cloned with HisTag tail at the C-terminus (Supplementary Figure S2). The obtained plasmids were transformed in *E. coli* BL21(DE3) chemically competent cells according to a standard heat shock protocol (Thermo Fisher). One transformed colony was used to inoculate 5 mL of selective medium with 50 μg/mL kanamycin and incubated at 37 °C with 250 rpm agitation. Once the pre-culture reached the mid-log phase (OD_600_ 0.4–0.6), it was expanded into a larger volume of fresh selective media (e.g., 1 L) in a flask. 0.5 mM IPTG was added to induce gene expression when the culture reached OD_600_ = 0.6. Bacterial cells were collected after 4 h by centrifugation at 4,000 xg for 10 min.

### Purification of histidine-tagged NGO_1985

The cell pellet was resuspended in Buffer A (20 mM NaH_2_PO_4_, 500 mM NaCl, 20 mM imidazole, pH 7.6), by adding 10 mL of buffer per one gram of wet cell mass. PMSF was added to 1 mM and DNase I-XT (NEB #M0570) to a final dilution of 1:10000. The suspension was sonicated using a Branson Sonifier 250 for 5 min at 50% time cycle, output level 6. The lysate was clarified by centrifugation at 38,800 xg for 40 min at 4 °C, and the supernatant was collected.

The protein was purified by metal affinity chromatography using an Äkta Pure 25 FPLC system (Cytiva). A 1 mL Ni-NTA column (Protein Ark) was pre-equilibrated with Buffer A, and the cleared lysate was applied to the column. The column was then washed with 25 mL of Buffer A and the protein was eluted with a linear gradient to 100% Buffer B (20 mM NaH_2_PO_4_, 500 mM NaCl, 500 mM imidazole, pH 7.6) over a 20 mL volume; 0.5 mL fractions were collected. The flow rate was 1 mL/min throughout.

The protein-containing fractions were pooled and concentrated using a centrifugal filter (Amicon 15, MW cut-off 10,000, Millipore) for size-exclusion chromatography (SEC). The sample was applied using a 0.5 mL loop to a Superdex 75 10/300 GL column (Cytiva) equilibrated with PBS, pH 7.4; 0.5 mL fractions were collected. The flow rate was 0.5 mL/min.

### Purification of the antibody–antigen complex

A mixture of the SEC-purified NGO_1985 and mAb_4 was applied to a Superdex 200 Increase 10/300 GL column (Cytiva) connected to the Äkta Pure 25 system in order to separate the antigen–antibody complex from free antigen. The running buffer was PBS, pH 7.4; sample loop volume 0.5 mL and flow rate 0.5 mL/min; 0.5 mL fractions were collected. Samples of the protein peak fractions were analyzed using SDS-PAGE.

### Size-exclusion chromatography with multi-angle light scattering (SEC-MALS)

Analytical SEC was performed using a multi-angle light scattering (MALS) detector with an embedded dynamic light scattering (DLS) module (MiniDAWN, Wyatt) and a differential refractive index (dRI) detector (Optilab, Wyatt) connected to the flow path of the FPLC system. Superdex 200 Increase 10/300 GL column (Cytiva), 0.5 mL/min flow rate and a 100 μL sample loop were used. The running buffer was 25 mM HEPES, 100 mM NaCl, pH 7.5). The results were analyzed using the ASTRA 8.2 software package (Wyatt).

### Differential scanning fluorimetry (DSF)

Samples of purified NGO_1985, mAb_4 and the mAb_4 – NGO_1985 complex were analyzed using a Prometheus NT.48 differential scanning fluorimetry (DSF) instrument (Nanotemper Technologies). Each protein sample was loaded into a Standard glass capillary (Nanotemper) and then measured using a temperature gradient from 20 to 95 °C at a rate of 2.0 °C/min. The results were analyzed (including melting curve inflection point determination) using the PR. ThermControl v.2.1.5 software (Nanotemper).

### Hydrogen-deuterium exchange mass spectrometry (HDX-MS) method

Epitope mapping of recombinant NGO_1985 antigen with antibody mAb_4 was performed by HDX-MS, comparing the amount of deuterium incorporated by NGO_1985 peptides in presence and absence of antibody. The antigen alone (30 pmol) or antibody/antigen complex (NGO_1985 /mAb 1/1 molar ratio) was incubated for 30 min at 25 °C. The labeling procedure, carried out at RT was initiated by adding deuterated PBS (pD of 7.3), reaching a deuterium excess of around 88% over five time points ranging from 15 s to 1,000 min (15 s, 1 min, 10 min, 100 min, 1,000 min) (Masson et al., 2019). Samples were quenched with a 3 M Guanidinium hydrochloride, 200 mM Phosphate buffer pH 2.32. Experimental replicates for statistical analysis were prepared for unbound and bound states at 15 s time point according to the recommendations for performing, interpreting and reporting hydrogen deuterium exchange mass spectrometry (HDX-MS) experiments (Masson et al., 2019). Samples were injected into a NanoAcquity UPLC with HDX technology (Waters Corporation) and digested on-line with a commercial pepsin column (Enzymate, Waters). The mass spectra of desalted and separated peptides were monitored on a Thermo Scientific Exploris 120 mass spectrometer equipped with an ESI source. Peptide identification was achieved by MS/MS analysis in Data Dependent Acquisition mode (DDA top 4) and data was processed using Peaks X software (Bioinformatics Solution). Only peptides within a mass tolerance of 10 ppm for the precursor ions and 0.02 Da for fragment ions were used for the following HDX-MS analysis. Differences in the amount of deuterium incorporated between the two states were analyzed using HDExaminer software (Sierra Analytics). For statistical analysis, three labeling reaction experiments of the antigen alone and in complex with the mAb were performed for the 15 s D2O exposure time point.

Analysis was performed by calculating a confidence interval (CI) based on the standard deviation (SD) in deuterium uptake at the time point performed in triplicates. For each state, the SDs were calculated using the root-mean-square as shown in Equation 1:


SD_state=√((∑▒(SD_i^2))/N) (1)

where 𝑁 is the total amount of peptides measured in replicate. The pooled SD, for the two states, was calculated using Equation 2:


SD_pool=√(〚SD〛_(stateA)^2+SD_(stateB)^2) (2)

The pooled SD was utilized to identify the 98% CI through Equation 3:


$$\mathrm{CI}=\bar{x}\pm 6.965\cdot (\mathrm{SD}\_\text{pool})/\surd n$$ (3)

Where 𝑥̅ is the assumed zero-centered average difference in deuterium uptake (𝑥̅=0), 6.965 is the t-value corresponding to a two-tailed distribution with two degrees of freedom, and 𝑛 is the analyzed sample size (set to 3 since the experiment was performed in triplicates).

### *In-silico* docking of mAb_4 and NGO_1985

*In silico* docking between the antibody mAb_4 and NGO_1985 was conducted using version 3 of the HADDOCK software (Giulini et al., 2025). A starting structure for NGO_1985 was generated via molecular modeling using the AlphaFold 3 approach (Abramson et al., 2024), generating five models used for further investigation. Similarly, the structure of the mAb_4 antibody was modeled with AbodyBuilder 3 (Kenlay et al., 2024), an artificial intelligence approach specifically designed for antibody structure prediction, which provided a highly confident estimation of the complementarity-determining regions (CDR). To guide the docking of the antibody–antigen complex, ambiguous interaction restraints (AIRs) (HADDOCK, 2025; Van Dijk et al., 2005) were generated. AIRs define regions on the interacting molecules expected to be at the binding interface, classifying residues as either *active* or *passive*. Active residues are those experimentally known or strongly predicted to be directly involved in the binding interface. Passive residues are solvent-accessible surface neighbors of active residues or other residues potentially involved in the interaction but not strictly required to be. For the antibody mAb_4 the paratope region was identified with Parapred (Liberis et al., 2018) and defined as active, while other CDR residues were defined as passive. HDX-MS data was used to define active residues in the NGO_1985 antigen structure, and all surface-accessible residues within 5 Å of these active sites were identified as passive. The HADDOCK protocol involved three stages: initial rigid-body docking (it0), semi-flexible refinement (it1), and explicit solvent refinement (water). In the rigid-body docking stage, 25,000 models were generated, following a semi-flexible refinement stage, and explicit solvent refinement. Throughout all stages (it0, it1, and water), the HDX-MS derived ambiguous interaction restraints were enforced with a tolerance of 2.0 Å. Additionally, unambiguous restraints were used to maintain the integrity of antibody chains. Following refinement, the resulting models were clustered using a Free Contact Correlation (FCC) cutoff of 0.75 Å and the top 5 models from the top 10 clusters were selected for further analysis.

### Molecular dynamics simulations

MD was performed using the OpenMM simulation toolkit (Fabritiis, 2024; Eastman et al., 2017) with the AMBER 14 force field parameter set (Maier et al., 2015; WashU, 2025). Explicit water molecules were included, and periodic boundary conditions were applied throughout the simulations. The method of particle-mesh Ewald was used for the calculation of the electrostatic potential energy. A no-bonded cutoff of 10 Å was implemented for long-range interactions. MD simulations were solvated (TIP3P model) and neutralized up to a final concentration of 0.15 M NaCl. An initial steepest descent minimization until the system’s potential energy gradient converges. Following minimization, the system underwent a gradual constant volume and temperature (NVT) with the velocity Verlet integrator with an integration time of 2 fs. The temperature was systematically raised by 5 K, starting from 5 K until it reached the target temperature of 310 K in 2 ps. Temperature control was maintained via the Langevin algorithm, and the SHAKE (Ryckaert et al., 1977) algorithm was used to fix water and protein bonds. Protein heavy atoms were harmonically restrained with a force constant of 100 kcal/mol^−1^ Å^−2^, for backbone and side-chain heavy atoms, respectively, to avoid artificial structural distortions. During NVT ensemble, temperature was gradually raised by 5 K every 0.1 ns starting from 5 K to reach the final temperature of 310 K. Next, an NPT ensemble (constant temperature and pressure) was maintained with a Langevin thermostat at 310 K and an anisotropic Monte Carlo barostat at 1 atm for 5 ns. During this time, the restraints on heavy atoms were gradually released. Finally, the production run was performed in an NTP ensemble for 100 ns, with frames saved every 0.1 ns. For the extended MD simulation, a production run of 1,000 ns was performed using the same simulation conditions.

### Binding free energy calculations (MM/GBSA)

Binding free energy was estimated using the Molecular Mechanics/Generalized Born Surface Area (MM/GBSA) method implemented in OpenMM (Eastman et al., 2017). Calculations were performed using the single-trajectory approach, where coordinates for the complex, receptor, and ligand are extracted from the same simulation frames, thereby neglecting the energetic penalty associated with conformational adaptation upon binding. Water molecules and ions were stripped from the trajectory prior to analysis. The potential energy of the system was calculated using the AMBER14 force field combined with the OBC2 implicit solvent model (Maier et al., 2015; Onufriev et al., 2004) to estimate the polar solvation free energy. The non-polar solvation term was estimated based on the solvent-accessible surface area (SASA) inherent to the GBSAOBC force implementation. No cutoff was applied to non-bonded interactions (NoCutoff method) to ensure accurate electrostatic evaluation. The approximate effective binding energy (ΔG_bind) was calculated according to the equation:


ΔG_bind=〈E_complex〉−(〈E_receptor〉+〈E_ligand〉)

where each term represents the sum of the gas-phase molecular mechanical energy ( E_MM) and the solvation free energy ( G_solv).

To account for the time-correlation inherent in molecular dynamics trajectories, statistical uncertainty was assessed using the block averaging method described by Flyvbjerg and Petersen (1989). The final binding energies are reported as the mean ± the block-corrected standard error of the mean (SEM).

### Trajectory analysis and structural stability

Post-simulation analysis was performed using custom Python scripts. Prior to calculation, trajectory frames were corrected for periodic boundary conditions by recentering the protein complex and ensuring molecular integrity. To assess structural drift, all frames were superposed onto the initial reference structure (energy-minimized starting pose) using $C_{\alpha }$ atoms. Conformational stability was monitored calculating the root mean square deviation (RMSD) of all $C_{\alpha }$ atoms to monitor overall equilibration:


$$\mathrm{RMS}D_{\mathrm{C\alpha}}(t)=\sqrt{\frac{1}{N}\sum_{i=1}^{N}{\left\|r_{i}(t)-r_{i}^{\mathrm{ref}}\right\|}^{2}}$$

To identify the most representative structural states, we employed Hierarchical Agglomerative Clustering implemented in the Scikit-learn library (Pedregosa et al., 2011) pairwise RMSD matrix was computed for the trajectory using protein $C_{\alpha }\;$ atoms. Clustering was performed using the average linkage algorithm with a spatial threshold of 0.15 nm. For each identified cluster, the representative structure (centroid) was defined as the “medoid” - the frame minimizing the average RMSD to all other members of the same cluster.

### Serum bactericidal assay (SBA) based on resazurin (R-SBA)

The assay was performed as previously described (Troisi et al., 2025). *Ng* strains were grown to mid-log phase and then incubated with the mAbs of interest in the presence of baby rabbit complement (BRC, Cedarlane). Bacterial viability was measured by adding resazurin and by recording the optical density at 405 nm with the Varioskan Lux microplate reader (Thermo Fisher Scientific).

### Visual opsonophagocytosis assay (vOPA)

The THP-1 opsonophagocytosis assay was performed according to the method described by Vacca et al. (2024). Briefly, THP-1 cells were cultured and differentiated with phorbol 12-myristate 13-acetate (PMA) prior to the addition of FA1090:sfGFP (Multiplicity of Infection – MOI - 40) that had been pre-incubated with monoclonal antibodies (mAbs). An unrelated IgG mAb was used as an isotype control. Cells were fixed with paraformaldehyde (PFA) and stained with anti-LOS 2C7 primary antibody, Alexa Fluor 568 secondary antibody (Thermo Fisher Scientific), CellMask™ Deep Red (Thermo Fisher Scientific), and DAPI (Thermo Fisher Scientific). Images were acquired using an Opera Phenix confocal microscope (Revvity). Image analysis was performed as previously described (Vacca et al., 2024).

### Transformation of *N. gonorrhoeae* FA1090 and analysis of transformants by colony PCR

A DNA fragment containing the NGO_1985 upstream and downstream homology arms and the kanamycin resistance cassette was amplified by PCR using the primers listed in Supplementary Table S2 and cloned in pUC19. Bacteria were streaked on Gc agar plates. On the following day, 30 μL of the DNA restriction fragment (concentration 20 ng/μl) or 10 μL of the PCR product (concentration 90 ng/μl) were combined with FA1090 resuspended in PBS. PBS only served as a negative control. After spotting the mixture onto a Gc agar plate with 1% Isovitalex, the plate was incubated for 6 h at 37 °C with 5% CO_2_. The bacterial spot was then streaked on a Gc agar plate containing 1% Isovitalex and 40 μg/mL kanamycin. After 24 or 48 h, the colonies were re-streaked onto a Gc agar plate that also contained 1% Isovitalex and 40 μg/mL kanamycin to validate transformation by colony PCR and generate glycerol stocks.

PCR master mix, either ReadyMix™ or JumpStart™ REDTaq, was used with specific primers (Supplementary Table S2). Colonies were picked with a sterile micropipette tip or a sterile toothpick and transferred into a PCR tube where they were resuspended in the PCR master mix. After PCR, 5 to 10 μL of each reaction were loaded into a 1% agarose gel for electrophoresis. Pictures of the gel were taken using the Thermo Fisher iBright™ FL1500 Imaging System instrument.

### RNA extraction and quantitative reverse transcription PCR (qPCR)

RNA was extracted from the wild-type FA1090 and ΔNGO_1985 strains using TRIZOL (Thermo Fisher). Bacterial pellets were resuspended in 1 mL TRIZOL and transferred to 2 mL tubes (Sarstedt), with 0.5 mL zirconia beads. Bacterial cells were disrupted by vortexing for 2 minutes. Beads were removed by a 30-s spin. The TRIZOL suspension was added to a MaxTract tube (Qiagen) and 200 mL chloroform was added. After vigorous shaking for 15 s, tubes were spun for 10 min at 4 °C at 12,000 x g to collect the upper (aqueous) phase which was transferred into a new tube. RNA was precipitated in sodium acetate 3 M DEPC pH 5.2 and 0.7 volumes of isopropanol. After centrifugation, the pellet was washed with 200 mL ethanol 70% and spun again for 15 min at 4 °C. The supernatant was removed, and the pellet dried and resuspended in DEPC-water. DNase treatment with Turbo DNase from Thermo Fisher was carried out according to the manufacturer’s recommendations. RNA concentration was measured by Nanodrop or Qubit, integrity was checked by agarose gel electrophoresis and finally stored at −80 °C.

Complementary DNA (cDNA) was synthesized from RNA using the Superscript Reverse Transcriptase enzyme (Thermo Fisher). The reaction mix included 500 ng of RNA, 1 μL random hexamer primers (Thermo Fisher), and 1 μL dNTPs (10 mM dNTP mix) in a final volume of 10 μL. Components were mixed and incubated at 65 °C for 10 min, then cold on ice adding: 2 μL 5x FSbuffer, 1 μL RNaseOUT (40 U/μL), 1 μL SuperScript IV, 1 μL 0.1 M DTT and 5 μL Nuclease-free water. The reverse transcription step was performed at 42 °C for 1 h. The enzyme was inactivated by incubation at 70 °C for 10 min. cDNA was stored at −20 °C or −80 °C until use. Primers for qPCR (Supplementary Table S2) were designed by using Primer3.^1^ SYBR® Green qPCR Mastermixes were used. Samples were run in a QuantStudio™ Real-Time PCR machine and analyzed by QuantStudio™ Real-Time PCR Software.

### Genomic DNA extraction, sequencing and whole genome analysis

*Ng* genomic DNA was prepared by using the QIAGEN® Genomic DNA kit. Bacteria were cultivated overnight at 37 °C in Gc liquid+ 1% Isovitalex. Cells were pelleted for 10 min at 4,000 xg and the bacterial pellet was processed according to the instructions of the manufacturer. Proteinase K treatment was included. TE buffer or nuclease-free water was used to elute the pure DNA from the spin columns. Genomic DNA sequencing was obtained at Eurofins Genomics by Illumina high-throughput sequencing technology, generating paired-end short reads. Raw sequencing reads were quality-checked and subsequently aligned to the reference genome corresponding to the reference strain. Read mapping was carried out using minimap2 (v2.26) (Li, 2018) employing the short-read preset optimized for accurate alignment of Illumina data. The resulting alignments were converted to BAM format, sorted, and indexed using SAMtools (Danecek et al., 2021). The mapped reads were inspected to confirm genome-wide coverage and to verify the presence of the intended gene knockout through the absence of read coverage and/or the presence of the expected deletion at the target locus.

## Results

### Selection of the anti-*ng* monoclonal antibody mAb_4

**Figure 1:**
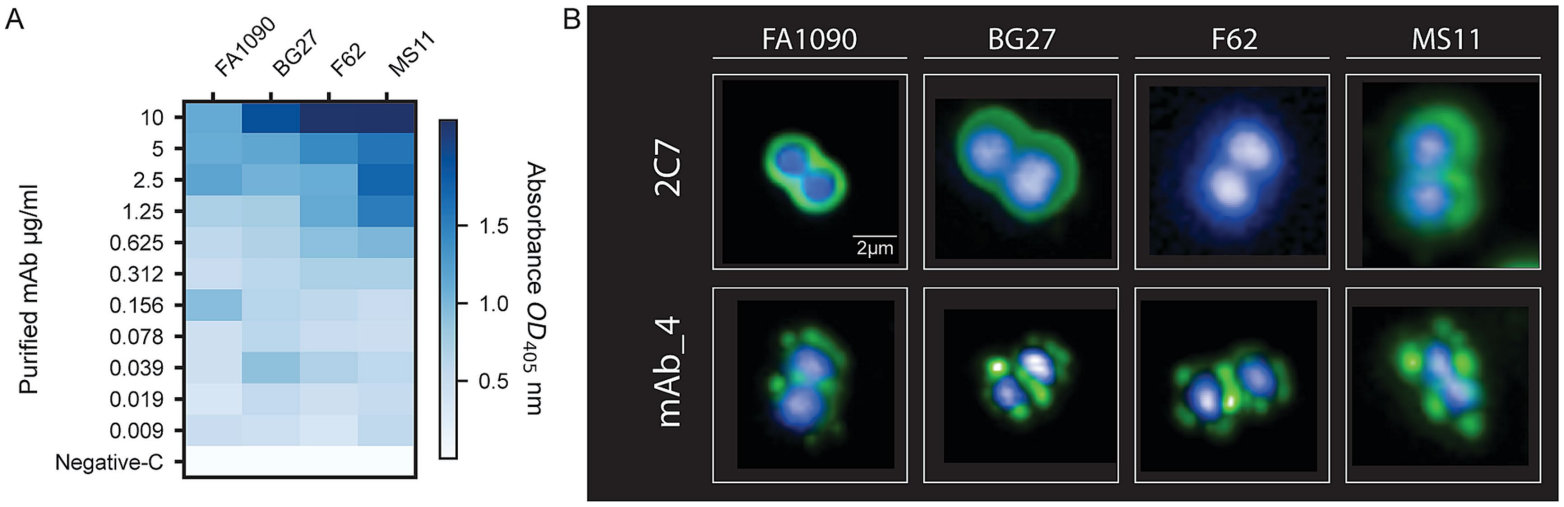
Binding of mAb_4 to *Ng* strains. **(A)** Assessment of the binding specificity of recombinantly produced mAb_4 to various *Ng* strains (indicated on top) by ELISA. Antibody concentration is indicated on the Y-axis. The negative control, indicated as “Negative C,” was represented by “no mAb” samples. The intensity of the binding signal is indicated by the heatmap whose scale is reported on the right. Experiments were performed in duplicate. **(B)** Qualitative confocal fluorescence microscopy study for the identification of mAb binding patterns to various *Ng* strains. Each image is representative of the majority of the bacterial population in the field of view. From left to right: immunostaining of FA1090, BG27, F62, and MS11 with the anti LOS mAb 2C7 (positive control) and mAb_4. The signal associated with the mAbs is in green while bacterial DNA is stained by DAPI (blue). Images were captured using a 63x objective on Opera Phenix. The scale bar is 2 μm.

In a previous study (Troisi et al., 2025) we isolated anti-*Ng* mAbs from healthy volunteers vaccinated with 4CMenB. This was achieved through single-cell sorting of MBCs specific for *Nm* MenB OMVs, followed by ELISA screening against *Ng* strains (Troisi et al., 2025). Among the 479 *Ng*-positive supernatants (Troisi et al., 2025) we selected mAb_4 for further characterization according to the pipeline illustrated in Supplementary Figure S1, given its cross-reactive binding profile to four different *Ng* strains, namely FA1090, BG27, F62, and MS11 (Figure 1A).

Further validation of mAb_4 interaction with *Ng* strains was performed using confocal fluorescence microscopy. While mAb 2C7 (Yamasaki et al., 1999), which targets the lipooligosaccharide (LOS) and was used as a positive control, uniformly bound all strains except F62, mAb_4 displayed a punctuated binding pattern across the four strains (qualitative observations in Figure 1B). Flow cytometry analysis confirmed binding of mAb_4 to the FA1090 strain but suggested that a minor fraction of the bacterial population expressed the antigen (Supplementary Figure S3).

The antibody was produced as a recombinant IgG1 molecule, using a scaffold incorporating the E430G mutation, known to promote hexamerization of the antibody and enhance functional activity upon binding to the antigen (de Jong et al., 2016).

### *In vitro* functionality of mAb_4

**Figure 2:**
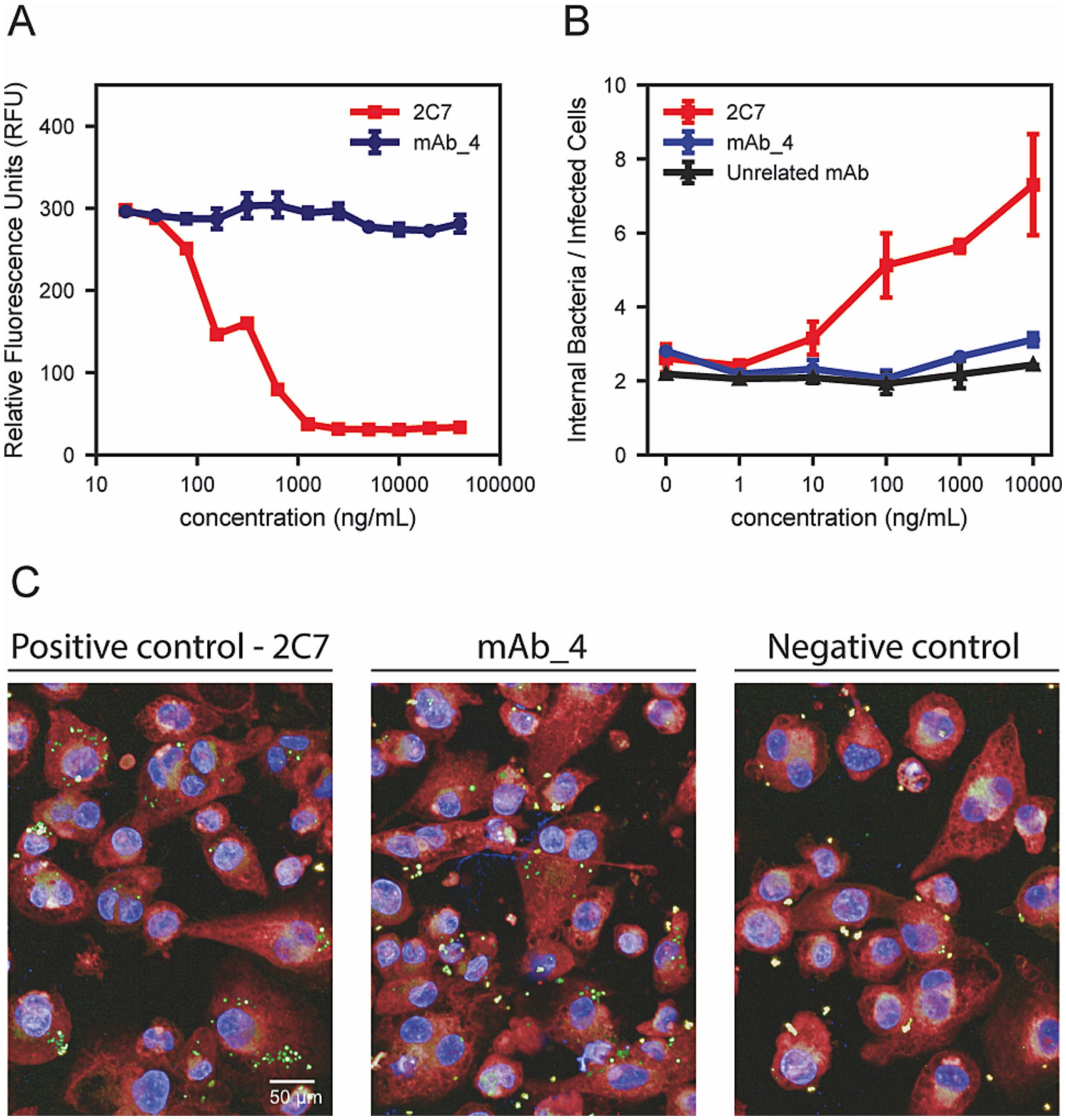
Evaluation of mAb_4 efficacy in complement-mediated killing and macrophage-mediated opsonophagocytosis. **(A)** Evaluation of mAb_4 efficacy in the R-SBA assay. The graph shows the average and standard deviation of relative fluorescence units (RFU, Y-axis) measured upon incubation of FA1090 with serial dilutions of mAb_4 (X-axis) in presence of baby rabbit complement. 2C7 was used as positive control. All samples were tested in triplicate. Two-way ANOVA statistical test was performed to compare each dose to the 2C7 condition: significant difference for 2C7 started at 100 ng/mL with *p* < 0.01 (higher doses showed increased significance with *p* < 0.0001). **(B)** Assessment of the opsonophagocytosis-promoting activity of mAb_4. The opsonophagocytosis-promoting activity of purified mAbs was measured in the presence of THP-1-derived macrophages. Differentiated THP-1 cells were incubated with FA1090, which was pre-opsonized by adding different amounts of mAbs 2C7 (positive control), mAb_4 and an unrelated IgG mAb which served as an isotype control. The plot shows the average and standard deviation of the number of internalized bacteria per cell. The different doses of mAbs tested are indicated on the X-axis, while the number of internalized bacteria per infected cell is reported on the Y-axis. Experiments were performed in triplicate. Two-way ANOVA statistical test was performed to compare each dose to the unrelated mAb condition: significant difference for 2C7 started at 100 ng/mL with *p* < 0.01 (higher doses showed increased significance with *p* < 0.0001). **(C)** Qualitative representative images of the opsonophagocytosis assay showing cells in red (Cell Mask), cell nuclei in blue (DAPI), internalized bacteria in green, and not internalized and adherent bacteria in yellow. The positive control is 2C7, and the negative control is an unrelated antibody. The scale bar is 50 μm.

The resazurin-based serum bactericidal assay (R-SBA) was employed to assess the bactericidal potency of mAb_4 against *Ng* strain FA1090 using baby rabbit complement (BRC) as an exogenous complement source. The anti-LOS mAb 2C7, included as a positive control, showed a concentration-dependent bactericidal effect with an IC_50_ of 0.169 μg/mL. In contrast, mAb_4 did not exhibit any bactericidal activity (Figure 2A).

Additionally, we assessed mAb_4’s ability to promote opsonophagocytosis by THP-1-derived macrophages using a confocal microscopy-based assay previously developed for this purpose (Vacca et al., 2024). The number of bacteria internalized by the macrophages was quantified by image analysis and the fold-change was compared to the average value observed in a negative control experiment performed using an unrelated mAb. Unlike the positive control, mAb_4 did not display a significant opsonophagocytosis-promoting activity (Figures 2B,C, where representative images are shown).

### Identification of mAb_4 target

**Figure 3:**
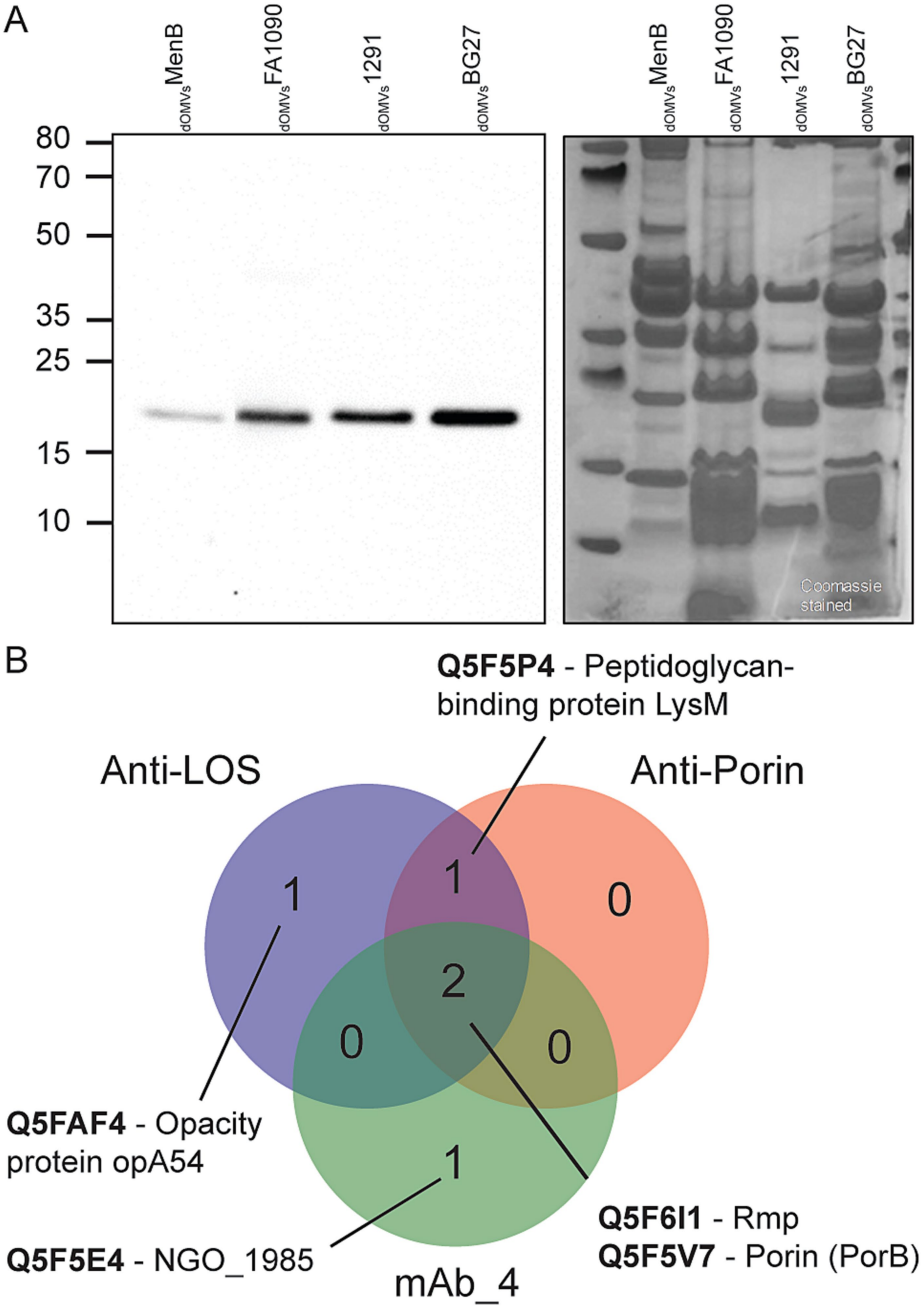
Identification and validation of NGO_1985 as the target of mAb_4. **(A)** Immunoblot obtained upon running OMV extracts from different *Ng* strains (FA1090, 1,291 and BG27) and from MenB and probed with mAb_4. The Coomassie-stained gel is shown on the right. **(B)** Results of immunoprecipitation experiments with mAb_4 on OMV extracts from FA1090. An anti-Porin mAb was used as a positive control. Enrichment of NGO_1985 was reported.

Despite the lack of functional activity in the *in vitro* assays described above, the peculiar binding pattern of mAb_4 stimulated our interest in the identification of its target antigen. Outer Membrane Vesicles (OMVs, Supplementary Figure S4) from *Ng* strains FA1090, 1,291 and BG27 were isolated and analyzed by immunoblotting using mAb_4. The antibody recognized a protein with an electrophoretic migration of approximately 20 kDa in all three *Ng* OMVs analyzed as well as in OMVs from the 4CMenB vaccine (Figure 3A), revealing cross-binding properties. Peptide mass fingerprint of the corresponding protein band identified NGO_1985, in agreement with the observed apparent molecular weight. Notably, NGO_1985 is the ortholog of *Nm* GNA_2091, a component of the 4CMenB vaccine (Vernikos and Medini, 2014; Seib et al., 2019).

To verify the above findings, we developed a protocol of immunoprecipitation using mAb_4, together with an anti-LOS and an anti-PorB mAb as controls (Troisi et al., 2025; Abcam, 2026; Thermo Fisher, 2026). OMV proteins were first solubilized with the detergent Nonidet P-40 (NP-40), and the mAbs were allowed to bind to their targets prior the purification of antigen/antibody complexes by Protein G. Immuno-precipitated antigens were then identified by mass spectrometry. While PorB and the closely related Rmp were identified in the three immunoprecipitations, NGO_1985 was detected only when mAb_4 was used, suggesting that NGO_1985 is the mAb_4 target (Figure 3B).

**Figure 4:**
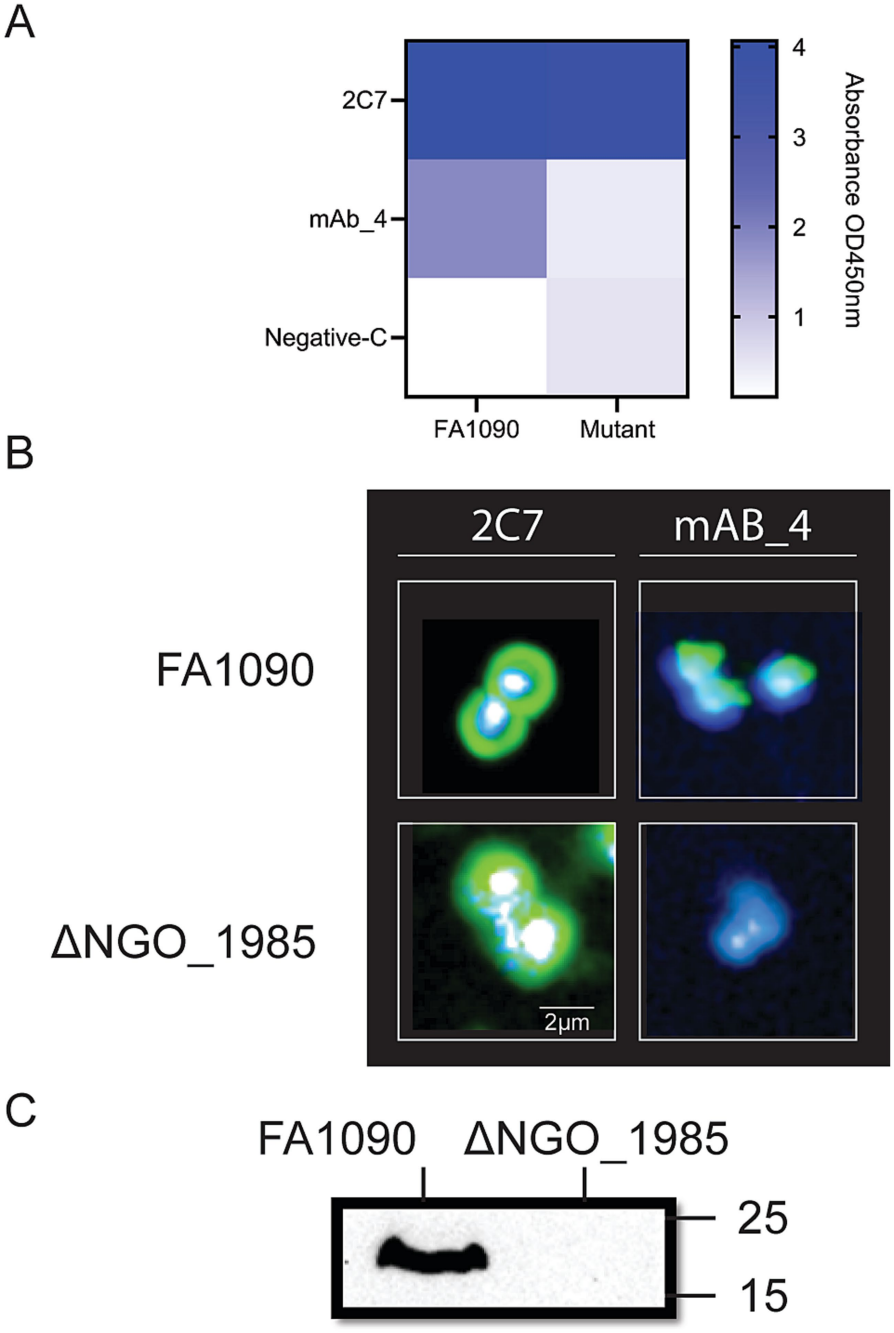
Loss of mAb_4 binding in the ΔNGO_1985 mutant confirms antigen specificity. **(A)** Results of ELISA experiments showing that mAb_4 does not bind to the NGO_1985 mutant strain. The positive control was anti-LOS mAb 2C7. The negative control (Negative-C in the figure) was represented by the “no mAb” condition. The heatmap legend is shown on the right. **(B)** Images from qualitative fluorescence confocal microscopy analysis generated upon incubation of FA1090 wild type and mutant strains with mAb_4 or with 2C7 (positive control). Bacterial DNA was stained with DAPI (blue). The images were acquired by using the Opera Phenix imaging system with a 63x objective. The scale bar is 2 μm. **(C)** Qualitative immunoblot on OMV extracts from FA1090 wild type and ΔNGO_1985 strains. mAb_4 was used to probe the membrane.

At last, an isogenic FA1090 NGO_1985 null mutant was created by inserting a kanamycin-resistance cassette in the gene encoding NGO_1985. Colony PCR, whole genome sequencing, and quantitative reverse transcription PCR were employed to verify the deletion (Supplementary Figure S5). ELISA (Figure 4A), confocal microscopy (a qualitative study is shown in Figure 4B), and immunoblot (qualitative analysis in Figure 4C) analyses performed with the mutant using mAb_4 were all negative, then definitively confirming NGO_1985 as the target of mAb_4.

### Characterization of the antibody–antigen complex

**Figure 5:**
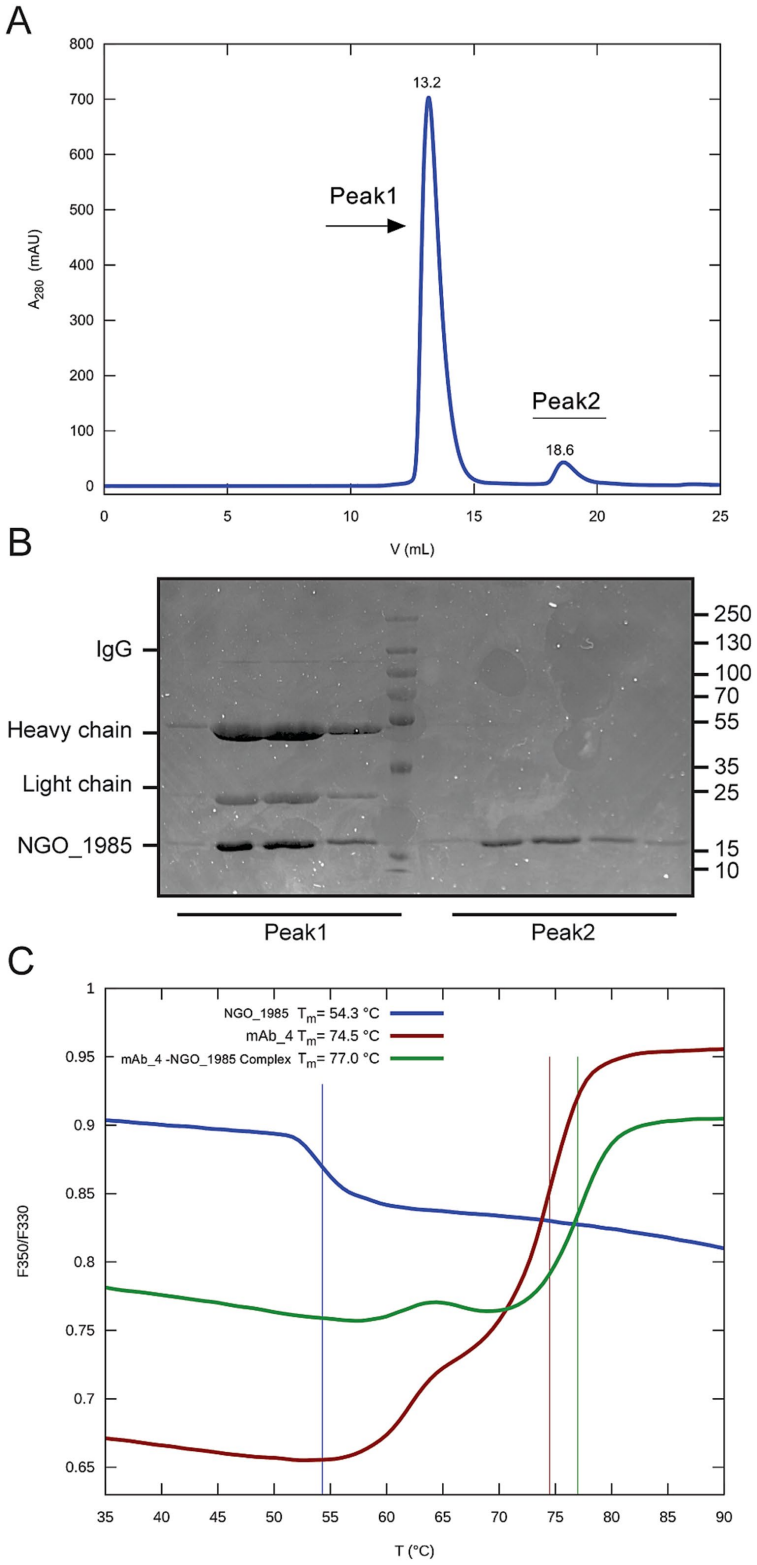
Investigations into the antibody–antigen complex structure. **(A)** Size-exclusion chromatography using a Superdex 200 Increase 10/300 GL column was run to separate the mAb_4 - NGO_1985 complex (Peak 1) from free NGO_1985 (Peak 2). **(B)** Materials collected from the two peaks were run in SDS-PAGE. 0.01 M 2-mercaptoethanol was used as a reducing agent. **(C)** Results of NanoDSF analysis of the mAb_4-NGO_1985 complex. The 350/330 ratio is related to the stability of the samples. NGO_1985 shows a blue-shifted transition (~54 °C), while mAb_4 melts at 74 °C; complex formation induces a red shift, increases Tm to 77 °C, and reduces aggregation.

NGO_1985 was also produced as recombinant protein to enable detailed characterization of the antibody–antigen complex. In SEC purification, NGO_1985 eluted at a volume corresponding to the monomeric form of the protein with a calculated molecular weight (MW) of 23.7 kDa (Supplementary Figure S6). When mAb_4 and the antigen were mixed in a 1:2 molar ratio and run on SEC, two well-separated peaks were observed, their elution volumes corresponding roughly to the calculated MW of the complex (173.7 kDa) and free NGO_1985 (Figure 5A). SDS-PAGE analysis of the peak fractions confirmed that the first-eluted peak consisted of the mAb_4–NGO_1985 complex and the later-eluted one of free NGO_1985 (Figure 5B). These SEC results suggest that the antibody binds to NGO_1985 in a molar ratio of 1:1, since the 1:2 ratio mixture contained a significant amount of free NGO_1985.

To further confirm these results, SEC-MALS characterization of the purified mAb_4 – NGO_1985 complex was conducted (Supplementary Figure S7). The observed molecular mass of the mAb_4 – NGO_1985 complex was 173.3 kD ± 0.1%, approximately matching the calculated mass of the 1:1 complex. This observation agrees with the results from the SEC analysis without MALS/DLS/dRI detectors (see above) and support the notion that mAb_4 binds NGO_1985 in a 1:1 molar ratio.

To study the effect of complex formation on thermal stability, the purified free NGO_1985, mAb_4 and the mAb_4 – NGO_1985 complex were studied using differential scanning fluorometry (DSF). All samples were in PBS (pH 7.4), the buffer that was used for SEC purification. In this method, the fluorescence emission of Trp residues is monitored at 330 (F330) and 350 nm (F350) over a temperature gradient. The thermal unfolding of a protein results in either a so called “red shift” in fluorescence emission (the F350/F330 ratio increases; if Trp residues are mainly buried at the beginning) or “blue shift” (F350/F330 ratio decreases; if Trp residues are exposed to solvent at start). In both cases, an estimate of the melting temperature (T_m_) of the protein can be determined from the plot of the ratio vs. temperature.

For NGO_1985, the F350/F330 ratio decreased (blue shift) during the thermal ramp used (20 — 95 °C), showing a melting transition with a T_m_ of 54.3 °C (Figure 5C). This was expected since in the published crystal structure of NGO_1985 (PDB entry 6XMV), the only tryptophan residue is located on the surface of the protein. For mAb_4 alone, a red-shift melting transition with a T_m_ of 74.5 °C was observed. In the mAb_4 – NGO_1985 complex, this melting transition shifts to a higher temperature, with a T_m_ of 77.0 °C, indicating that, as expected, the binding of the ligand stabilizes the antibody.

### Epitope characterization of anti- NGO_1985 mAb_4

The interaction of NGO_1985 with mAb_4 was investigated by Hydrogen/Deuterium exchange Mass Spectrometry (HDX-MS) and by *information-driven* docking and molecular dynamics.

**Figure 6:**
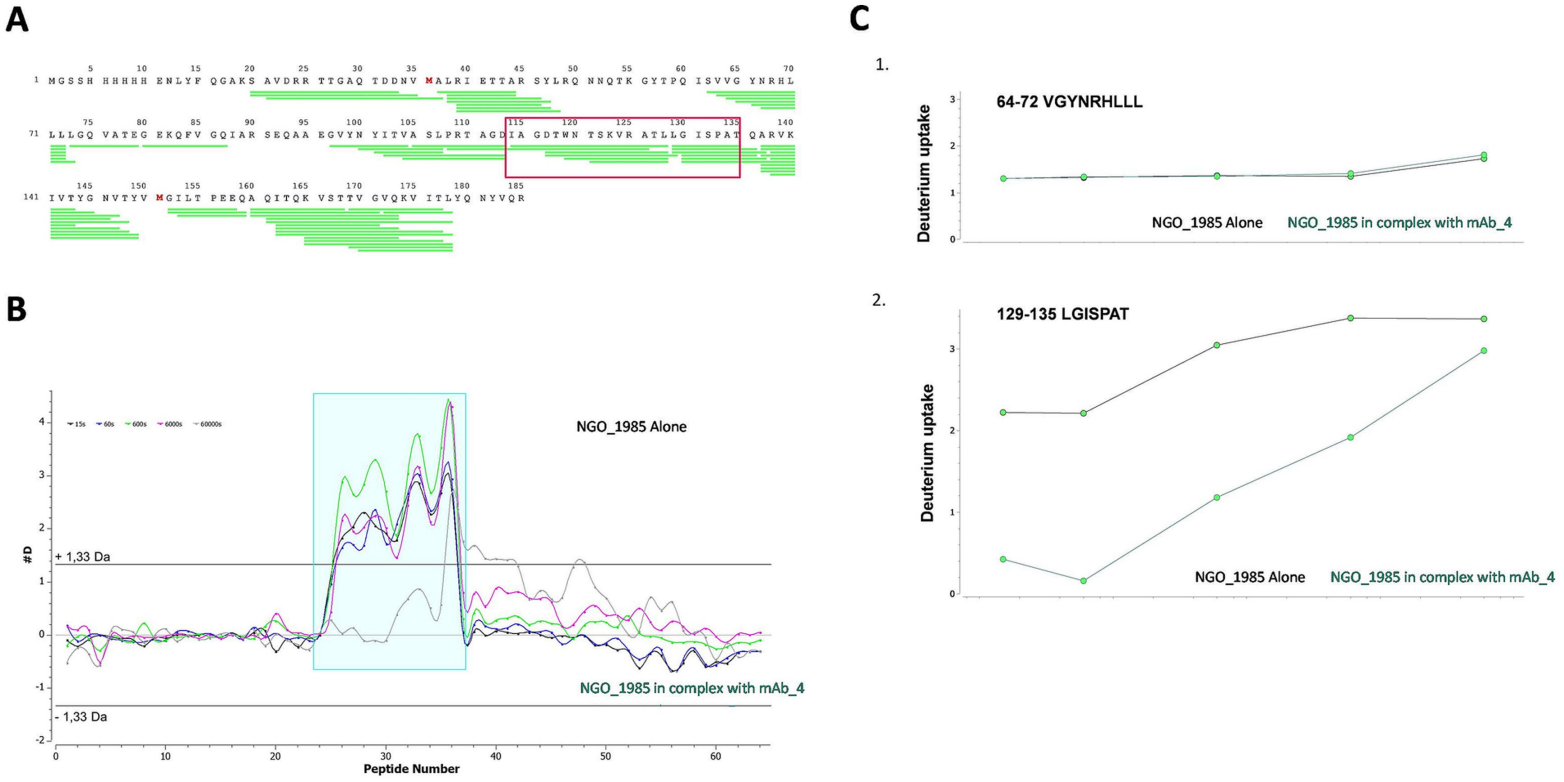
Region Ile114-Thr135 was recognized by mAb_4 in HDX-MS experiments. **(A)** Peptide map of the recombinant NGO_1985 protein. The green lines represent the 64 peptides monitored during the HDX experiment, covering 73% of the protein sequence with a redundancy of 3.6. The oxidized Met residues are reported in red. **(B)** Residual plot of the HDX-MS epitope mapping of mAb_4 on recombinant NGO_1985. The plot shows the differences in deuterium uptake (Y-axis) for the 64 identified peptides in the absence and presence of mAb_4 across time points ranging from 15 s to 1,000 min. Peptides are arranged on the X-axis from the N-terminus to the C-terminus. Positive values on the Y-axis (blue box) indicate regions protected from deuterium incorporation in the presence of mAb_4, defining an epitope located within residues I114 - Thr135. Dotted lines represent the 98% confidence interval. **(C)** Representative deuterium uptake profiles for two peptides: One outside the epitope (Val64-Leu79) (C.1) and one within the epitope (residues Leu129-Thr135) (C.2).

HDX-MS analysis has been accepted as the most effective approach for rapidly providing near-complete information on epitope structure (Jethva and Gross, 2023; Malito et al., 2013). The recombinant form of NGO_1985 was labeled with deuterium in presence or absence of mAb_4. The difference in deuterium incorporation was monitored on 64 peptides, covering 73% of protein sequence with a redundancy of 3.6 (Figure 6A). Upon binding of the mAb a reduction in deuterium incorporation was observed on 11 overlapping peptides, located in residues Ile114 to Thr135 (Figure 6B). In Figure 6C, representative deuterium uptake profiles for two peptides are shown: one located outside the epitope (residues Val64-Leu79) in which no difference in deuterium incorporation between the two states along the four time points were observed and one located within the epitope in which a major incorporation of deuterium was observed in NGO_1985 alone with respect to the corresponding complex peptide (residues Leu129-Thr135).

**Figure 7:**
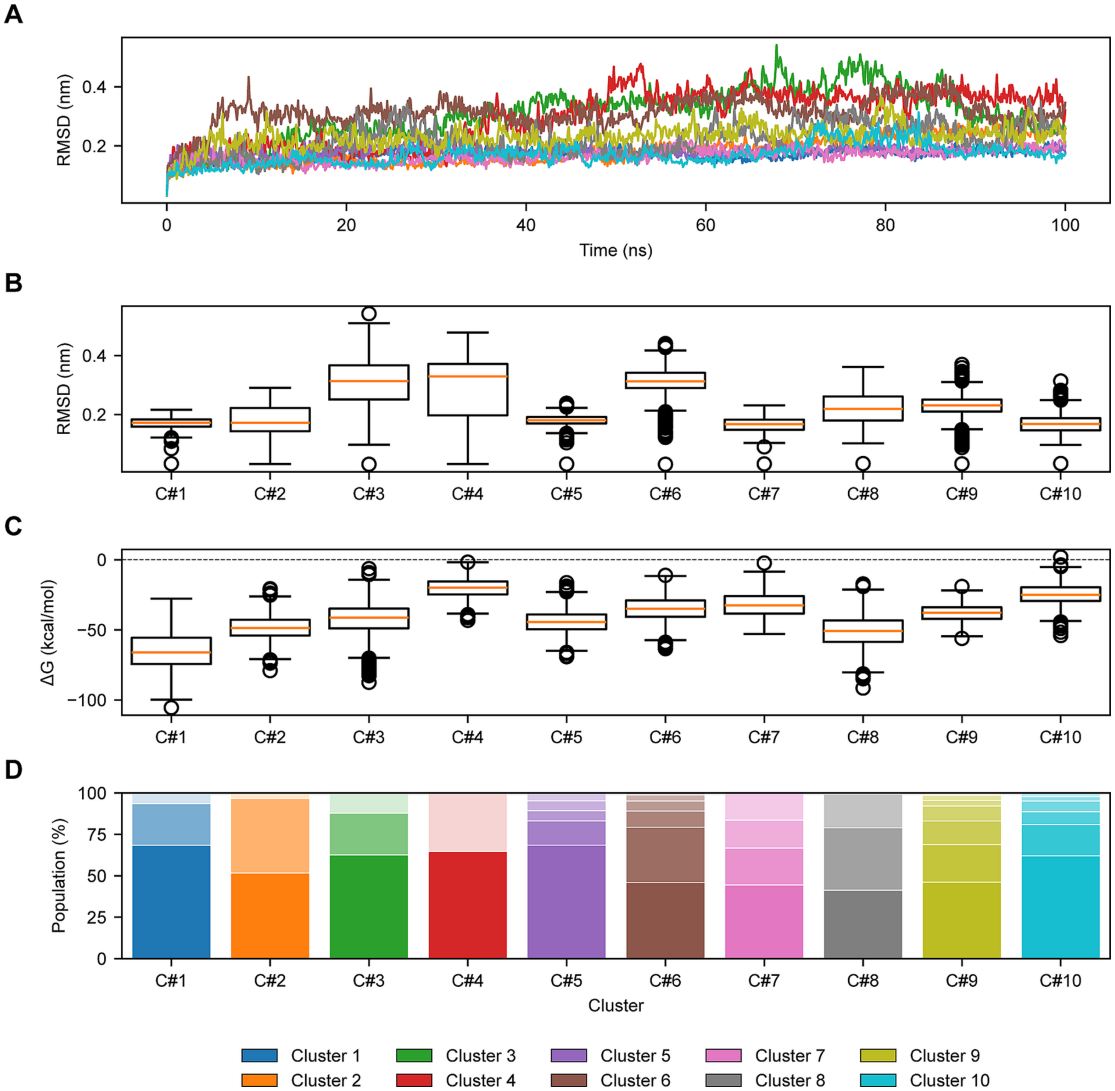
Molecular dynamics screening of the top 10 HADDOCK-generated antibody–antigen complexes. **(A)** Backbone RMSD trajectories of the antigen (aligned to the antibody) over 100 ns for the ten highest-scoring HADDOCK clusters. **(B)** Distribution of RMSD values for each pose. Pose 1 (C#1) and Pose 7 (C#7) exhibit the lowest median deviation and interquartile range, indicating high structural rigidity. **(C)** Binding free energy (dG) estimated using Molecular Mechanics/Generalized Born Surface Area (MM-GBSA). Lower values indicate stronger affinity. Pose 1 and Pose 8 display the most favorable binding energies, though Pose 8 shows significant outliers. **(D)** Conformational stability analysis via cluster distribution. The vertical bars represent the population of structural clusters within each trajectory (RMSD cutoff 0.15 nm). Pose 1 is characterized by a single dominant conformational state (>68% occupancy), whereas other low-energy candidates (e.g., Pose 2, Pose 8) show fragmented populations indicative of structural toggling or drift.

To date, no experimental structure of NGO_1985 in complex with an antibody has been reported by X-ray crystallography, NMR, or cryo-EM. Furthermore, no residue-resolved interface mapping has been described, and no predictive structural models of the interaction are available. The antibody–antigen interaction was therefore investigated *in silico* by integrating deep-learning structure prediction, information-driven docking, and molecular dynamics simulations. The antibody and antigen structure were predicted using ABodyBuilder3 (Kenlay et al., 2024) and AlphaFold3 (Abramson et al., 2024), respectively. Next, the structures were docked using a data-informed HADDOCK protocol, by confining the epitope definition to the region identified by HDX-MS and the antibody paratope. This protocol returned the top ten ranked binding conformations, based on their docking scores. To identify the most stable and probable conformation, 100 ns all-atom molecular dynamics simulations were performed for each of the ten poses (Supplementary Table S3). As shown in Figure 7, the candidates exhibited divergent dynamic behaviors. Poses 3 through 6 and Pose 10 displayed high structural fluctuations (RMSD > 0.3 nm, Figures 7A,B) and weak binding affinities (dG > −40 kcal/mol, Figure 7C), leading to their exclusion. The remaining candidates were evaluated based on a convergence of energetic and entropic stability. While Pose 8 exhibited competitive binding energies (approximately −54 kcal/mol), the clustering analysis (Figure 7D) revealed significant conformational heterogeneity, with the population split between two distinct states (41% vs. 37%). This suggests that Pose 8 represents an unstable or transient association rather than a locked complex. In contrast, Pose 1 emerged as the most robust candidate, combining the most favorable binding energy (dG = −60.7 kcal/mol) with structural stability, maintaining a single dominant conformation for >68% of the simulation.

### Structural analysis corroborates the experimental results

**Figure 8:**
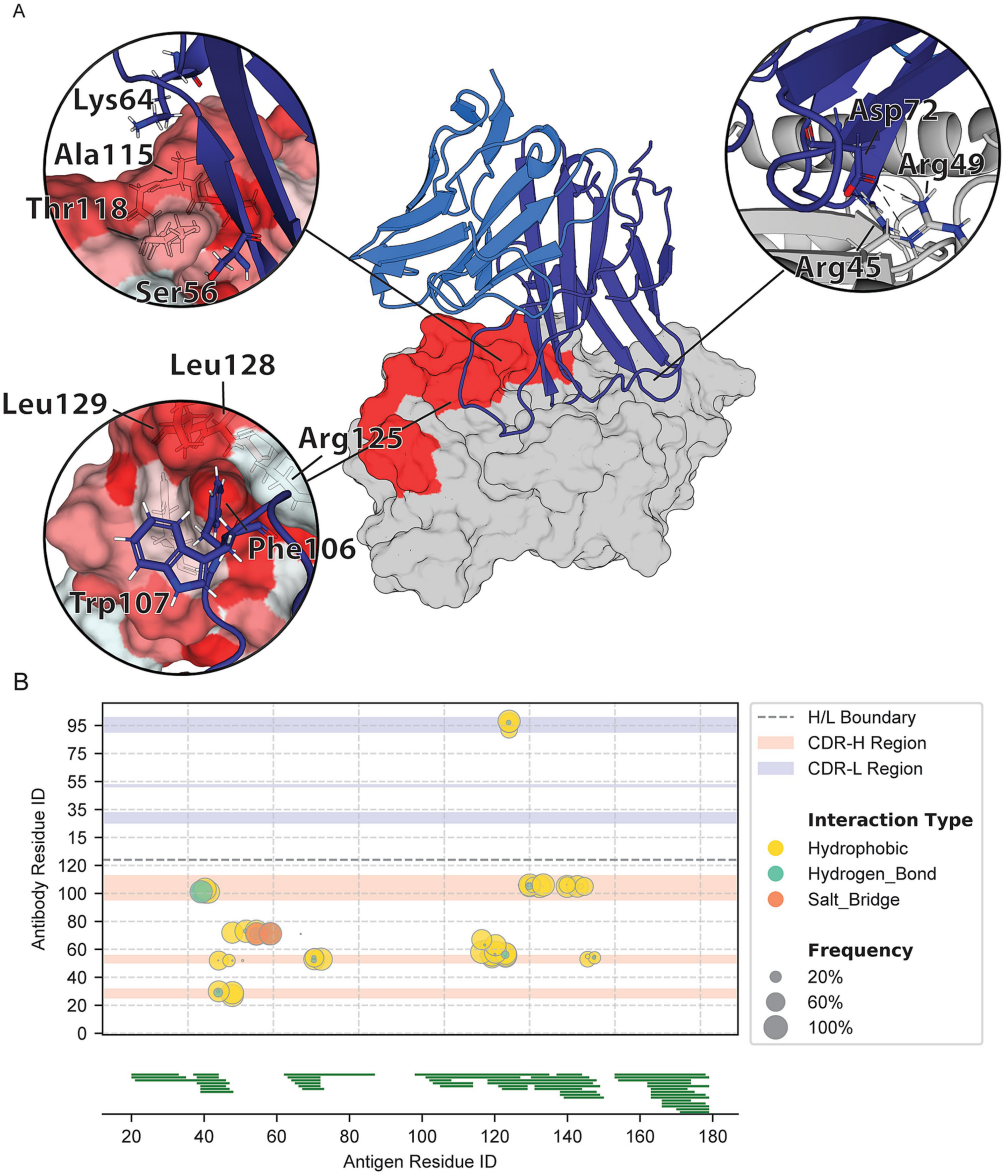
Structural basis of antibody–antigen interactions. **(A)** Detailed view of stabilizing interactions. Bottom left: The hydrophobic core formed by aromatic residues Phe106 and Trp107 of the heavy chain inserting into the antigen hydrophobic pocket; top left: CDR-H2 residues of the heavy chain are involved in a hydrophobic interaction with antigen residues Ile114 through Thr118; top right: The stabilizing salt-bridge network connecting Asp72 of the antibody framework to Arg45 and Arg49 of the antigen. **(B)** Contact map summarizing antibody–antigen interactions identified during molecular dynamics simulations. Each dot represents an interaction between a specific antibody–antigen residue pair. Dot colors indicate the interaction type, while dot size is proportional to the interaction frequency across the trajectory. Antibody complementarity-determining regions (CDRs) are highlighted as semi-transparent horizontal bands, colored by chain (heavy chain in red, light chain in blue). The dashed horizontal line denotes the boundary between heavy and light chains. On the x axes the peptide map of the antigen shown below the contact map, indicating the positions of overlapping peptides used in the experimental epitope mapping. The x-axis corresponds to antigen residue numbering and is aligned with panel A to facilitate direct comparison between interaction hotspots and experimentally probed regions.

Structural analysis of the Pose 1 trajectory revealed a binding interface dominated by the heavy chain, with the light chain contributing only minor peripheral contacts (residues Phe218-Gly224). The stabilization of the complex appears to be driven by a combination of a dense hydrophobic network and critical electrostatic anchors (Figure 8). The core of the interface is defined by the heavy chain CDR-H3 and CDR-H2 loops. Specifically, a cluster of aromatic residues - including Tyr59, Phe106, and Trp107 - forms a robust hydrophobic clamp around the antigen surface. Phe106 and Trp107 engage in extensive contacts with a C-terminal antigen patch comprising Arg125, Leu128, Leu129, and Gln136 (Figure 8A, bottom left). This hydrophobic interaction is further supported by CDR-H2 residues (His53-Lys64), which interface with antigen residues Ile114 through Thr118 (Figure 8A, top left). Complementing this hydrophobic core, the complex is anchored by a significant electrostatic network involving the framework and CDR regions. Most notably, Asp72 forms a salt-bridge with Arg45 and Arg49 on the antigen (Figure 8A, top right). This interaction likely restricts the conformational freedom of the antigen N-terminus. Additional stability is provided by hydrogen bonds at the periphery of the interface, specifically between Ser30-Asn34, Ser56-Thr118, and Asp102- Leu129. Overall, the docking model corroborates the HDX data, as the primary hydrophobic interface lies within the 114–135 segment (Figure 8B).

## Discussion

A retrospective case–control study identified a significant association between receipt of the meningococcal OMV vaccine at a sexual health clinic and a reduced risk of gonorrhea infection (Paynter et al., 2019). To explore the molecular mechanisms behind cross-protection between *Nm* and *Ng*, we isolated mAbs against *Ng* from individuals vaccinated with the meningococcal vaccine 4CMenB. Among these antibodies, mAb_4 was demonstrated to cross-react with several *Ng* strains. Based on the distinct binding pattern exhibited by mAb_4, we decided to search for the cognate surface antigen.

Despite lacking affinity and binding kinetics measurements, our study revealed that mAb_4 binds to a specific protein on different *Ng* strains, which is called NGO_1985 and is the ortholog of *Nm* GNA_2091, a fusion antigen in the 4CMenB vaccine that contributes to its stability and immunogenicity (Zielke et al., 2014; Tsolakos et al., 2014). Previous efforts have provided a foundation for pinpointing potential *Ng* antigens (Li et al., 2021). NGO_1985 has previously been referred to as BamG in two gonococcal studies; however, its role within the Bam complex has not been mechanistically demonstrated, and it differs from the recently characterized BamG in *Bacteroidetes* (Silale et al., 2025).

One objective of this work was to map the epitope of NGO_1985 targeted by mAb_4. Successful integration of *in silico* and HDX-MS approaches allowed the identification of the specific region of NGO_1985 involved in the interaction. Comparative analysis revealed strong conservation within *Neisseria* species and partial conservation across other Gram-negative bacteria. The key residues, including aspartate and tryptophan, are maintained, suggesting functional constraint (Holliday et al., 2009) and supporting their importance for enzymatic activity. Notably, this motif overlaps with mAb_4 recognized epitope. Since a significant portion of the antigen is periplasmic and not entirely surface-exposed, the NGO_1985 epitope mapped here could guide future NGO_1985 - based vaccine development. Ultimately, our work successfully identified a mAb from 4CMenB-vaccinated individuals that recognizes a fusion protein component (GNA_2091 or NGO_1985) known for contributing to protection in animal models (Tsolakos et al., 2014).

The inability of the mAb to induce complement-dependent bactericidal activity or to opsonize the bacteria suggests that antibodies with different affinity for the target or different complement fixation capacity might be required. Alternatively, other mechanisms different from bacterial killing or opsonophagocytosis might be involved *in vivo*. Our speculation is that the observed cross-protection between *Ng* and *Nm* likely requires antibodies against multiple targets or alternative anti-bacterial mechanisms such as inhibition of bacterial adherence to epithelial cells. Consistent with this complexity, recent randomized clinical evidence from the GoGoVax trial showed that the 4CMenB vaccine did not reduce gonorrhea incidence compared with placebo in high-risk individuals (NSW, 2026; CIDRAP, 2026). Indeed, studies by Semchenko et al. (2019) and our own (Troisi et al., 2025) suggest that protection is associated with a polyclonal immunity directed against the LOS, the porin, and other proteins like NGO_1985 discovered here. Nevertheless, the antibody characterized in this study holds potential for exploring cocktail therapies and for use as a diagnostic or biotechnology tool. Of interest, mAb_4 facilitated the development of immunoprecipitation/mass spectrometry (IP/MS) analysis protocols for identifying unknown antibody targets, especially the less abundant ones.

In conclusion, this study demonstrated that, despite the limitations mentioned above (i.e., limited strain panel, lack of biological activity in the assays employed, lack of affinity and binding kinetics data), in addition to the most obvious and abundant antibody targets like LOS and porins, vaccination with 4CMenB elicits an immune response against proteins embedded in the outer membrane. Given the conservation of these protein antigens in the *Neisseria* genus, they can be considered for the rational design of broadly protective vaccines or for optimizing novel and much needed medications.

## Data Availability

The MS data presented in the study are deposited in the PRIDE repository with accession number PXD079527.

## Footnotes

1
https://primer3.ut.ee/
