# Supplementary material for: Discovery and characterization of an anti-Neisseria gonorrhoeae NGO_1985 monoclonal antibody and cognate antigen
Source: Front Microbiol. 2026 Jun 24;17:1830193. doi: 10.3389/fmicb.2026.1830193 (PMC13341933; doi:10.3389/fmicb.2026.1830193)
Supplement: Supplementary file 1 [file Data_Sheet_1.PDF]

SUPPLEMENTARY INFORMATION for the manuscript

Discovery and characterization of an anti-*Neisseria gonorrhoeae* NGO_1985 monoclonal antibody and cognate antigen

Pardis Mokhtary[1,2], Samuele Stazzoni[1§], Eleonora Marini[1], Marco Troisi[1], Tomi T. Airenne[3,4], Mikko Huhtala[3,4], Giuseppe Maccari[5§], Lucia Eleonora Fontana[6], Nathalie Norais[6], Laura Tinti[7], Vittoria Cicaloni[7], Laura Salvini[7], Monica Fabbrini [6], Tiina A. Salminen[3,4], Emanuele Andreano[1§], Claudia Sala[1§] & Rino Rappuoli[8*]

[1] Monoclonal Antibody Discovery Laboratory, Fondazione Toscana Life Sciences, Siena, Italy.

[2] Department of Biotechnology, Chemistry and Pharmacy, University of Siena, Siena, Italy.

[3] Structural Bioinformatics Laboratory, Biochemistry, Faculty of Science and Engineering, Åbo Akademi University, Tykistökatu 6A, 20520 Turku, Finland.

[4] InFLAMES Research Flagship Center, Åbo Akademi University, 20520 Turku, Finland.

[5] Data Science for Health Laboratory, Fondazione Toscana Life Sciences, Siena, Italy.

[6] GSK, Siena, Italy.

[7] Mass Spectrometry Unit, Fondazione Toscana Life Sciences, Siena, Italy.

[8] Fondazione Biotecnopolo di Siena, Siena, Italy.

[§] Current address: Fondazione Biotecnopolo di Siena, Siena, Italy

* Corresponding authors:

Email: rino.rappuoli@biotecnopolo.it

SUPPLEMENTARY TABLES

Table S1. *Neisseria gonorrhoeae* strains used in this study.

	FA1090	BG27	F62	MS11
**Isolation site**	Lab strain	Clinical isolate	Lab strain	Lab strain
**Source**	ATCC	University of Bristol, UK	ATCC	University of Massachusetts USA
**Serum susceptibility**	Resistant	Resistant	Sensitive	Sensitive
**Genetic island (GGI)***	Absent	Unknown	Absent	Present

*GGI: Gonococcal genetic island constitutes the first major discriminating factor between *Ng* and the other *Neisseria*.

Table S2. List of primers used in this work.

Name	Sequence (5'-3')	Restriction enzyme/ Product length (bp) and Features
	NGO_1985 homology Arms Up & Down	
NGO1985-upArm-Fw	CTATCGATGAGCTCTGCCGACGCGCAACACTTCG	*Sac*I
NGO1985-upArm-Rv	ATCCGCGGTACCCGGTGTGTGGTTTGGGTTTCATCGG	*Kpn*I, *512 bp*
NGO1985-downArm-Fw	GCTCAGGGATCCCCAAAACTACGTCCAACGC	*Bam*HI
NGO1985-downArm-Rv	GATTCGAAGCTTGGGCGTTTGTGGGCAGTACG	*Hind*III, *522 bp*

	Primers used to verify in-frame deletion mutants	
NGO1985-check-Fw<br><br>NGO1985-check-Rv	GCCGAGTCAGCGTTGGACGTAG<br><br>GGAAGTCCCCGATGAAACCCAAACC	*522 bp*
	Kanamycin (Neo) cassette	
Neo-Fw<br><br>Neo-Rv	ATCCGCGGTACCCGGTGGGGTCCCCAATAATTACGAT<br><br>GCTCAGGGATCCCAATTAATTATTAGAAAAATTCATCCAGC	*KpnI*<br><br>*BamHI*<br>*816 bp*
	qPCR-Primers	
NGO_1985,FW<br><br>NGO_1985,RV	CTACCTGCGCCAAAACAACC<br><br>GGGAGGCGACGGTAATGTAG	*182 bp*
NGO_1984,FW<br><br>NGO_1984,RV	GCTCATCTGTATCACCGCCATC<br><br>GTTTGGAGGGTTTTGTCGCC	*84 bp*
NGO_1983,FW<br><br>NGO_1983,RV	CTACCCCAAATCCTGCTGCAC<br><br>GGAGACTTTGCCGACGGTAAAC	*163 bp*

Table S3. Summary of pose analysis and structural stability

Pose	Clusters No.	dG	dG SEM	rmsd
1	3	-64.71	3.753	0.17 ± 0.019
2	3	-48.52	1.465	0.183 ± 0.046
3	3	-42.95	6.931	0.309 ± 0.086
4	2	-20.34	2.764	0.297 ±0.091
5	6	-44.28	3.94	0.18 ± 0.019
6	7	-35.07	1.75	0.312 ± 0.048
7	5	-32.08	1.778	0.166 ± 0.024
8	4	-50.73	5.126	0.22 ± 0.054
9	8	-38.01	1.886	0.231 ± 0.038
10	7	-24.53	2.016	0.17 ± 0.035

Supplementary Figures

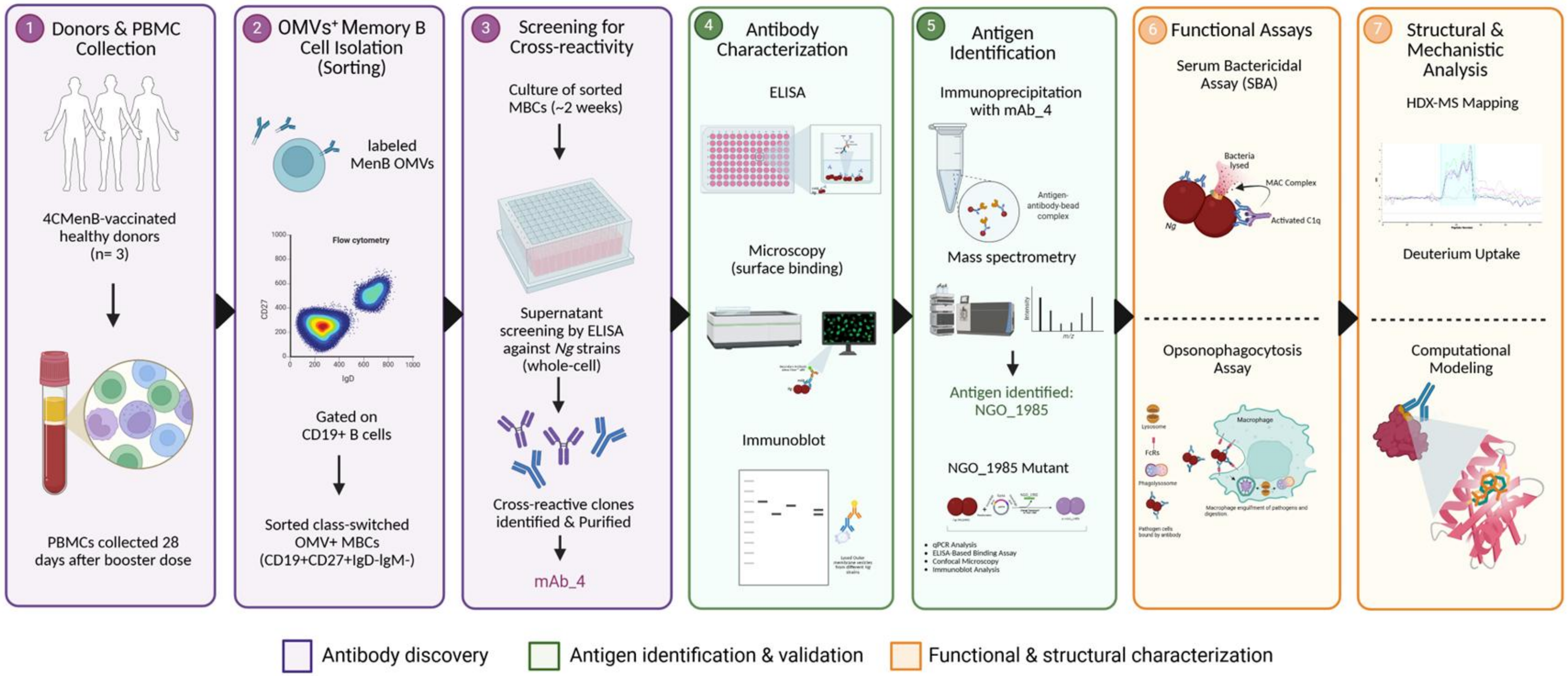

Figure S1. Overview of the antibody discovery and characterization workflow. 1. Peripheral blood mononuclear cells (PBMCs) were collected from 4CMenB-vaccinated donors. 2. OMV-specific memory B cells were isolated by flow cytometry. 3. Antibody-containing supernatants were screened for cross-reactivity against *Neisseria gonorrhoeae*, leading to the identification of mAb_4. 4. Antibody binding was characterized using ELISA, immunoblotting, and microscopy. 5. The target antigen was identified by immunoprecipitation and mass spectrometry as NGO_1985. To validate NGO_1985 as the target, a knockout strain was generated and characterized by qPCR, ELISA binding assays, microscopy, and immunoblot analysis. 6. Functional activity was assessed by serum bactericidal and opsonophagocytosis assays. 7. Structural insights were obtained through HDX-MS and computational modeling.

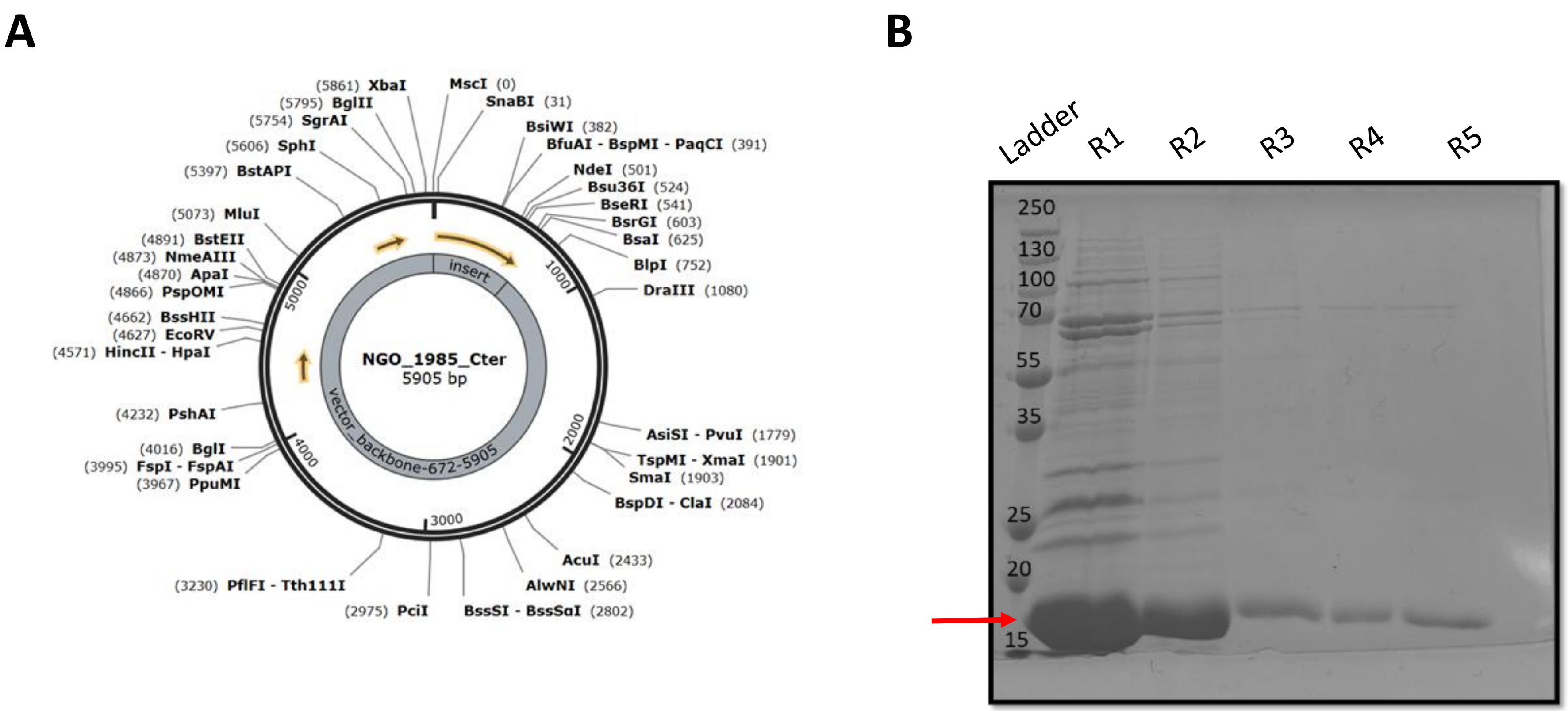

Figure S2. Expression of C-terminal-His-tagged NGO_1985 and purification. A. pET28a was used for cloning the coding sequence of NGO_1985**,** with 6xHis at either the N-terminus or the C-terminus. B. After the first step of purification (nickel affinity purification by FPLC), five fractions were collected, labeled R1 to R5, and analyzed on SDS-PAGE. The red arrow indicates NGO_1985.

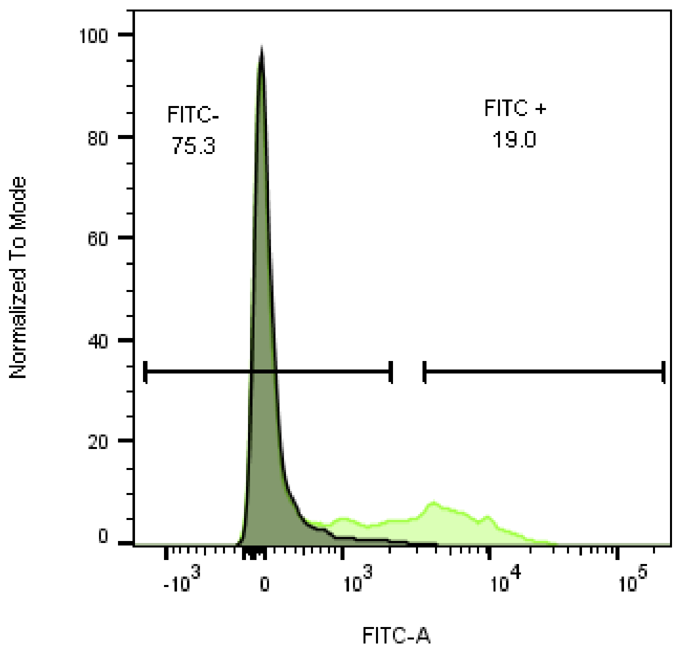

Fig S3. Binding characterization by flow cytometry for mAb_4 against FA1090. The graph presents flow cytometry histogram analyses, where the deep green histogram represents the negative control, and the light green histogram corresponds to the binding of mAb_4 targeting a specific antigen on the FA1090 strain.

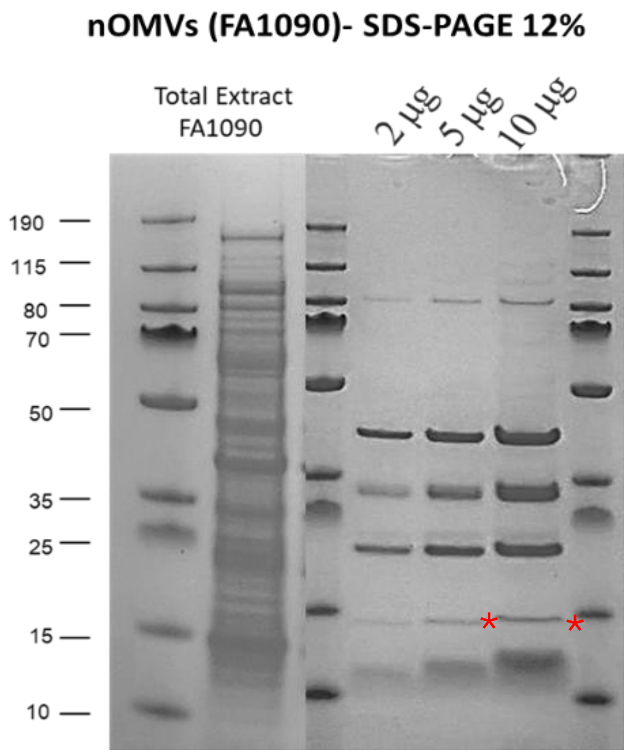

Fig S4. Coomassie-stained SDS-PAGE of FA1090 OMVs. Different amounts of OMVs were loaded on the gel and demonstrated different enrichment levels of the proteins present in the vesicles. The spot of interest is indicated between two asterisks.

A

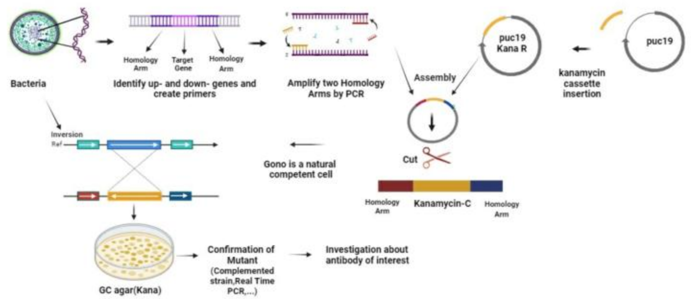

B

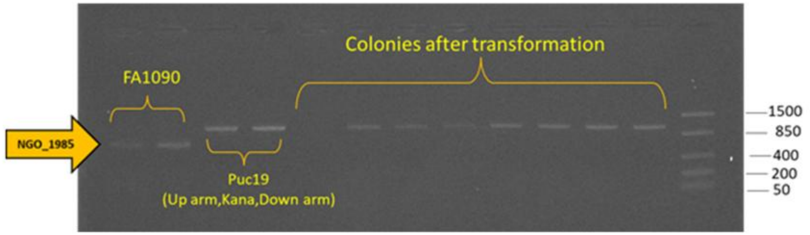

C

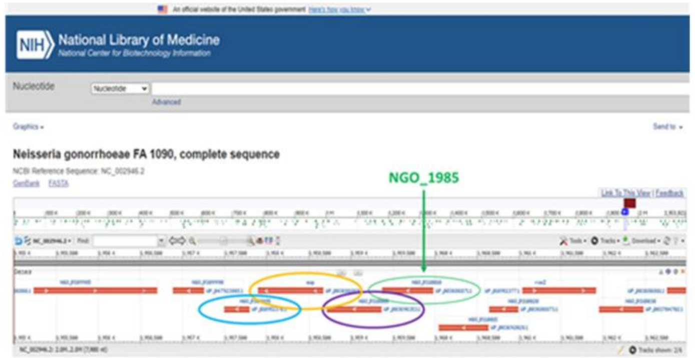

D

Gene	Relative expression (ΔΔCT method)	Conclusion
NGO_1985	N/A	The gene has been knocked out
NG0_1984	1.8	NGO_1984 is 1.8 times more expressed in the mutant as compared to the wild type
NGO_1983	1.4	NGO_1983 is 1.4 times more expressed in the mutant as compared to the wild type

E

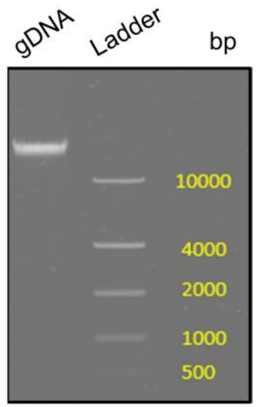

F

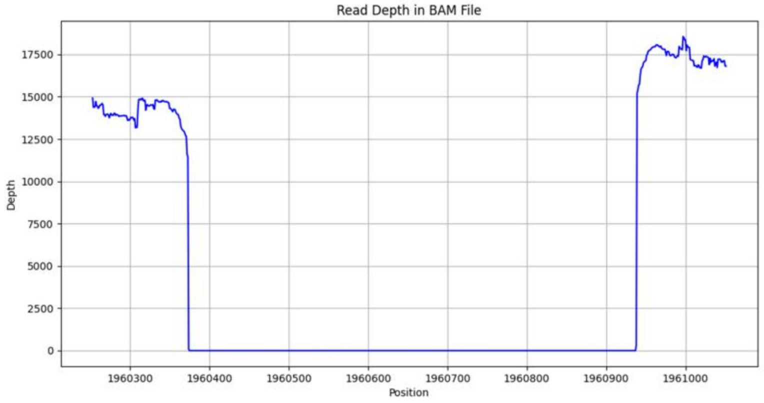

Fig S5. Generation of the NGO_1985 mutant strain.
Caption on next page

Fig S5. Generation of the NGO_1985 mutant strain. A. To validate NGO_1985 as the target of mAb_4, a knockout mutant in the encoding gene was constructed by allelic exchange. The NGO_1985 DNA sequence and the upstream and downstream regions were downloaded from the FA1090 genome sequence (GenBank accession number AE004969). Primers for homology arm amplification were designed and used to generate a construct where the kanamycin resistance cassette was flanked by NGO_1985 upstream and downstream DNA regions. FA1090 was then transformed with the linear construct and transformants analyzed by colony PCR. B. Colonies that had undergone successful allelic exchange showed the same size of 955 bp. C. Genetic analysis of the FA1090 NGO_1985 locus suggested that the gene might be part of a transcriptional unit that included NGO_1984 and NGO_1983**.** To examine the potential polarity effect resulting from the deletion of gene NGO_1985, a quantitative PCR analysis was conducted on both the ΔNGO_1985 strain and the FA1090 WT strain. Specific primers were designed (Table S1) for NGO_1985, NGO_1984, and NGO_1983 while *recA* was chosen as the housekeeping reference gene. D. Upon calculating the relative expression of the three genes of interest using the ΔΔCT method, the NGO_1985 gene was confirmed to be successfully knocked out in the mutant strain. E. To further validate the ΔNGO_1985 strain, genomic DNA was extracted. The image shows agarose gel electrophoresis of the genomic DNA extracted from ΔNGO_1985. F. Mapping of the Illumina sequencing reads to the FA1090 reference genome demonstrates the deletion of NGO_1985 in the mutant strain. Position in the genome is on the X-axis while read counts are on the Y-axis. Results of the alignment of the Illumina reads to the FA1090 reference genome confirmed the successful knockout of the gene NGO_1985 and its replacement by the kanamycin resistance cassette. Importantly, the kanamycin resistance cassette was inserted into the NGO_1985 locus only and was not found in any other genomic locus of the ΔNGO_1985 strain.

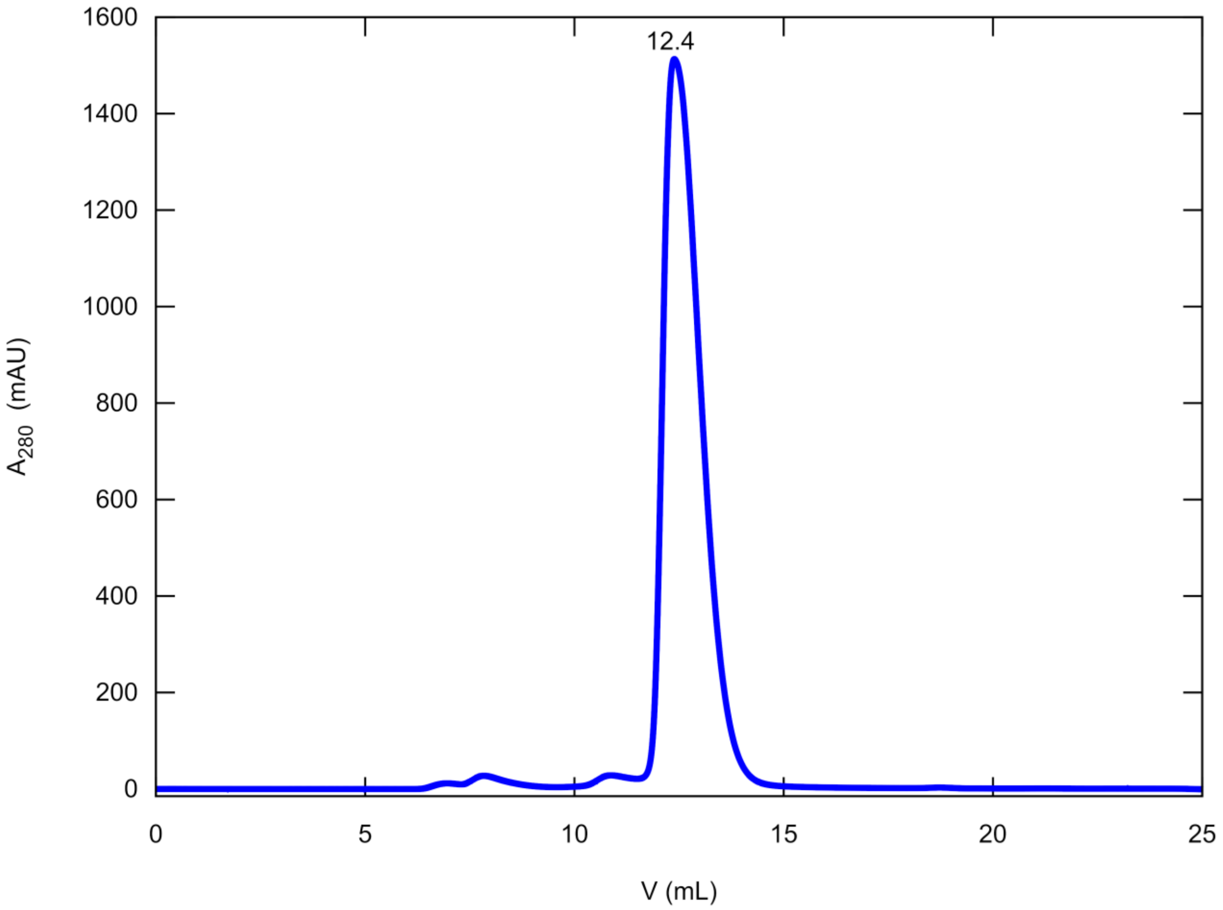

Figure S6. SEC chromatogram of the metal-affinity-purified recombinant NGO_1985. A Superdex 75 10/300 GL column, PBS (pH 7.4) as the running buffer, and a 0.5 mL sample loop were used. Note that the SEC column is different from that in Figure 5 and in Figure S7, and therefore the elution volumes are not comparable. Here, the elution volume of the main peak, Ve = 12.4 mL, corresponds to a molecular weight of 20.9 kDa according to the calibration curve of this column (Cytiva calibration standards; data not shown). This agrees with the calculated molecular weight of the monomeric recombinant NGO_1985, 23.7 kDa, within the accuracy of the used method.

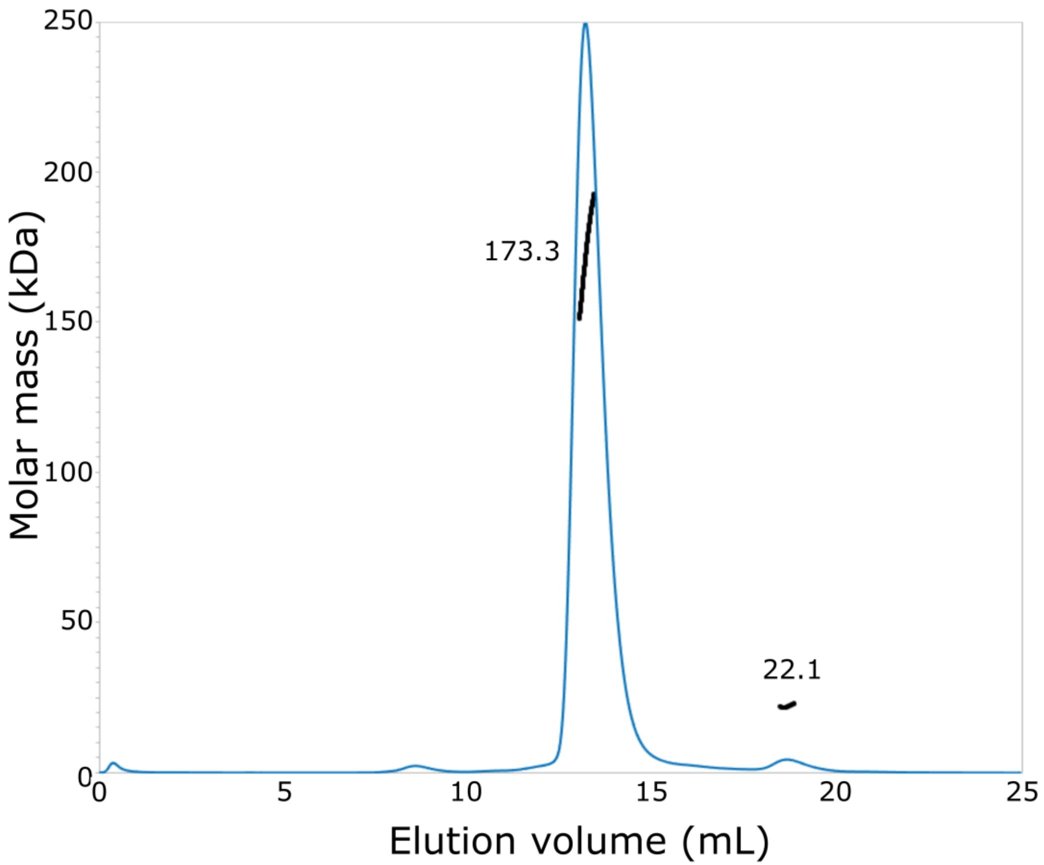

Figure S7. SEC-MALS analysis of NGO_1985-mAb4 complex. A Superdex 200 Increase 10/300 GL column was used. The light scattering signal is shown as a blue curve. Molar masses (kDa) are shown for the complex and free NGO_1985 in numbers and as black curves (distribution within the peaks).
